# Microglia directly associate with pericytes in the central nervous system

**DOI:** 10.1002/glia.24371

**Published:** 2023-03-30

**Authors:** Gary P. Morris, Catherine G. Foster, Jo‐Maree Courtney, Jessica M. Collins, Jake M. Cashion, Lachlan S. Brown, David W. Howells, Gabriele C. DeLuca, Alison J. Canty, Anna E. King, Jenna M. Ziebell, Brad A. Sutherland

**Affiliations:** ^1^ Tasmanian School of Medicine, College of Health and Medicine University of Tasmania Hobart Tasmania Australia; ^2^ Wicking Dementia Research and Education Centre, College of Health and Medicine University of Tasmania Hobart Tasmania Australia; ^3^ Nuffield Department of Clinical Neurosciences University of Oxford Oxford UK; ^4^ Global Brain Health Institute Trinity College Dublin Ireland

**Keywords:** Alzheimer's disease, capillary, in vivo two‐photon microscopy, microglia, pericyte

## Abstract

Cerebral blood flow (CBF) is important for the maintenance of brain function and its dysregulation has been implicated in Alzheimer's disease (AD). Microglia associations with capillaries suggest they may play a role in the regulation of CBF or the blood–brain‐barrier (BBB). We explored the relationship between microglia and pericytes, a vessel‐resident cell type that has a major role in the control of CBF and maintenance of the BBB, discovering a spatially distinct subset of microglia that closely associate with pericytes. We termed these pericyte‐associated microglia (PEM). PEM are present throughout the brain and spinal cord in NG2DsRed × CX_3_CR1^+/GFP^ mice, and in the human frontal cortex. Using in vivo two‐photon microscopy, we found microglia residing adjacent to pericytes at all levels of the capillary tree and found they can maintain their position for at least 28 days. PEM can associate with pericytes lacking astroglial endfeet coverage and capillary vessel width is increased beneath pericytes with or without an associated PEM, but capillary width decreases if a pericyte loses a PEM. Deletion of the microglia fractalkine receptor (CX_3_CR1) did not disrupt the association between pericytes and PEM. Finally, we found the proportion of microglia that are PEM declines in the superior frontal gyrus in AD. In summary, we identify microglia that specifically associate with pericytes and find these are reduced in number in AD, which may be a novel mechanism contributing to vascular dysfunction in neurodegenerative diseases.

## Main Points

- Microglia dynamically associate with pericytes in the brain.
- Microglia can contact the basement membrane covering pericytes.
- The proportion of microglia associated with pericytes and capillaries is reduced in Alzheimer's disease.

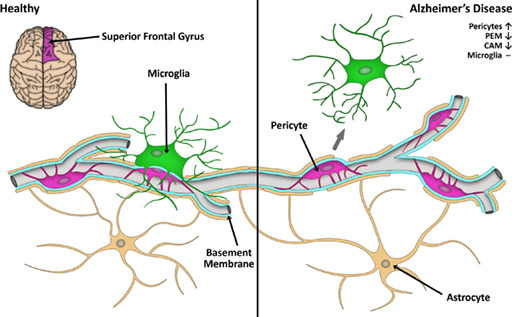

## 1 INTRODUCTION

Microglia are highly ramified, dynamic cells that contribute to homeostatic functions including structural plasticity, synaptic plasticity, neurite formation, myelination and vasculogenesis (Prinz et al., 2021; Werneburg et al., 2017; Wright‐Jin & Gutmann, 2019). Microglia are also central players in the innate immune system of the central nervous system (CNS), rapidly responding to challenges to the CNS and playing important roles in the initiation of inflammation, removal of debris and promotion of various CNS recovery mechanisms (Prinz et al., 2021). Microglia are known to have a close relationship with the vasculature during CNS development and in adulthood (Barón & Gallego, 1972; Cammermeyer, 1965; Cuadros et al., 1993; Lassmann et al., 1991; Rezaie et al., 1997), with these microglia having been variously termed “vascular satellites” (Barón & Gallego, 1972; Oemichen, 1978; Rio Hortega, 1920), “perivascular microglia” (Barón & Gallego, 1972; Graeber & Streit, 1990; Oemichen, 1978), “juxtavascular microglia” (Grossmann et al., 2002; Mondo et al., 2020; Oemichen, 1978), “vessel‐associated microglia” (Haruwaka et al., 2019), and more recently “capillary‐associated microglia” (CAM) (Bisht et al., 2021). Given a subset of microglia reside adjacent to vessels, microglia may play a role in blood vessel function and maintenance. In support of this, recent studies have illustrated that ablation of microglia triggers capillary dilation (Bisht et al., 2021) and impairs evoked blood flow changes following stimulation (Bisht et al., 2021; Császár et al., 2022). The mechanisms governing their contribution to these functions are not yet clear, but it is possible they influence blood flow indirectly through pericytes (Bisht et al., 2021; Kisler et al., 2021; Mills et al., 2021).

Pericytes are cells embedded on the walls of capillaries throughout the body (Brown et al., 2019; Sutherland, 2021) and are well characterized as modulators of cerebral blood flow (CBF) (Hall et al., 2014). However, little is known about the relationship between microglia and pericytes. Early reports confused the two cell types due to their close spatial relationship (Risau & Wolburg, 1990) and a collection of evidence suggests pericytes may differentiate into microglia (Barón & Gallego, 1972; Özen et al., 2014; Sakuma et al., 2016). More recent reports detail that there is extensive contact of pericytes by microglia in the developing (Hattori et al., 2022) and adult brain (Császár et al., 2022) and in the retina (Mills et al., 2021). Outside of these findings, it is not known how often microglia and pericytes are spatially associated, or whether any spatial association between the two cell types may serve a functional purpose.

As many as 80% of individuals diagnosed with Alzheimer's Disease (AD) have some level of vascular pathology in the brain including microinfarcts and cerebral amyloid angiopathy (Toledo et al., 2013). Both impaired CBF and blood–brain‐barrier (BBB) breakdown are also reported to occur early in the pathogenesis of AD (Steinman et al., 2021). Although the etiology of vascular alterations in AD is unknown, increasing evidence has implicated pericytes in blood flow dysfunction, neurodegeneration and cognitive decline in AD (Nortley et al., 2019; Sagare et al., 2013). In the human brain, several studies have suggested pericytes are susceptible to death in brain regions affected by AD (Halliday et al., 2016; Kirabali et al., 2020; Sengillo et al., 2013), which would hypothetically have detrimental consequences for blood flow regulation. Similarly, a growing body of literature has implicated microglia dysfunction as a key feature of AD pathogenesis. Several sporadic AD risk genes are highly expressed in microglia (Jonsson et al., 2013; Schwabe et al., 2020), maladaptive microglia over‐strip synapses in mouse models of AD (Hong et al., 2016) and microglia adopt specific phenotypes in AD (Keren‐Shaul et al., 2017). Given the role pericytes and microglia may play in the pathogenesis of AD, any breakdown in the functional association between these two cell types may also influence AD pathogenesis.

In this study, we first investigated the spatial relationship between microglia and pericytes in the adult mouse brain and spinal cord. In doing so, we discovered a subset of microglia that dynamically interact with pericytes. We have named these cells pericyte‐associated microglia (PEM). Using in vivo two‐photon laser scanning microscopy (2PLSM), we found that PEM can maintain their position adjacent to pericytes for at least 28 days in the somatosensory cortex and are present at all levels of the capillary vascular tree. In post‐mortem human AD tissue, we found a lower proportion of microglia that were PEM in the frontal cortex compared to controls, despite an increase in the number of pericytes. These results point towards a close relationship between microglia and pericytes that, if disrupted, could contribute to the vascular pathogenesis of neurodegenerative diseases, including AD.

## 2 MATERIALS AND METHODS

### 2.1 Animals

All animal procedures were approved by the Animal Ethics Committee, University of Tasmania (A18606, A18608 and A23735) and conformed with the Australian National Health and Medical Research Council (NHMRC) Code of Practice for the Care and Use of Animals for Scientific Purposes, 2013 (8th Ed.). All results are reported in accordance with the ARRIVE guidelines (Percie du Sert et al., 2020). Hemizygote NG2DsRed transgenic mice (The Jackson Laboratory stock #008241) were backcrossed onto a C57BL/6J background and crossbred with CX_3_CR1^GFP/GFP^ transgenic mice (The Jackson Laboratory stock #005582, C57BL/6J background) to produce NG2DsRed × CX_3_CR1^+/GFP^ or NG2DsRed × CX_3_CR1^GFP/GFP^ mice. A total of 32 mice were used throughout the study. Mice were group housed in Optimouse caging on a 12/12 h light/dark cycle with ad libitum access to food and water, with acclimation for ≥7 days.

### 2.2 Tissue collection, processing and labeling

#### 2.2.1 Tissue collection

12‐week‐old NG2DsRed × CX_3_CR1^+/GFP^ or NG2DsRed × CX_3_CR1^GFP/GFP^ mice were given a lethal intraperitoneal injection of pentobarbitone (300 mg/kg) and immediately transcardially perfused with heparinised PBS followed by 4% paraformaldehyde (PFA; pH 7.4). Whole brains were prepared for cryosectioning as previously described (Courtney et al., 2021).

#### 2.2.2 Blood vessel labeling and immunohistochemistry

For vessel labeling one section per brain was mounted onto a microscope slide (Dako, Cat# K802021‐2), air dried and permeabilised in PBS with 1% Triton X‐100 for 20 min at room temperature (RT). Sections were incubated overnight at 4°C with isolectin GS‐IB4 (IB4, 1:100; Invitrogen, Cat# I32450) in PBS with 1% Triton X‐100. Sections were washed with PBS (3 × 5 min) and treated with Trueblack (Biotium, Cat# 23007) according to the manufacturer's instructions. Sections were washed in PBS (3 × 5 min), rinsed in distilled water, briefly airdried, and coverslipped with Prolong Gold with DAPI (Life Technologies, Cat# P36935).

Sections for Aquaporin‐4 (AQP4) labeling were slide mounted (Dako, Cat# K802021‐2), permeabilised in PBS with 0.3% Triton X‐100 for 40 min, washed in PBS (3 × 5 min) and incubated with serum free protein block (Dako, Cat# X090930‐2) for 1 h at RT. Sections were incubated overnight at 4°C with guinea pig‐anti‐AQP4 antibody (1:250; Synaptic Systems, Cat# 429004, RRID: AB_2802156) in antibody diluent (Dako, Cat# S080983‐2). Sections were rinsed with PBS (3 × 5 min) and incubated in goat‐anti‐guinea pig 647 (1:1000; ThermoFisher, Cat# A21450) in antibody diluent for 2 h at RT. Sections were washed in PBS (3 × 5 min), treated with Trueblack, washed in PBS (3 × 5 min), rinsed in distilled water, briefly airdried and cover slipped with Prolong Gold with DAPI.

### 2.3 Imaging of CAM/PEM in the mouse brain

DAPI ± IB4 stained NG2DsRed × CX_3_CR1^+/GFP^ and NG2DsRed × CX_3_CR1^GFP/GFP^ tissue was imaged using a VS120 Virtual Slide System (Olympus, Japan). Sections used for CAM/PEM analysis were imaged at 20× magnification across five focal planes, spaced 2 μm apart, using the extended focal imaging setting. Optimum exposure times for DAPI (excitation [Ex]: 388 nm; emission [Em]: 448 nm), DsRed (Ex: 576 nm; Em: 625 nm), GFP (Ex: 494 nm; Em: 530 nm) and IB4 (Ex: 640 nm; Em: 690 nm) were determined manually and kept consistent for all images in each cohort.

Confocal stacks of IB4 and AQP4 labeled NG2DsRed × CX_3_CR1^+/GFP^ tissue were spaced at 1.0 or 0.5 μm increments, using an inverted Ti Eclipse microscope (Nikon, Japan) equipped with a CSU‐X1 spinning disk scanner (Yokogawa Electric Corporation, Japan). Images were acquired with a 100× (1.40) oil objective (Nikon, Japan) with filters for DAPI (405/445), FITC (488/525), TRITC (561/615), and CY5 (640/705) and analyzed using NIS‐Elements AR 5.02.00 (Nikon, Japan). Images were exported as OME TIFFs and NIS‐Elements AR 5.02.00 was used to create 3D reconstructions.

### 2.4 Cranial window implantation and in vivo two‐photon imaging

Cranial windows were implanted in nine 12‐week‐old NG2DsRed × CX_3_CR1^+/GFP^ mice as described previously (Tang et al., 2021), with modification; windows were implanted without a titanium bar glued opposite the window and were placed over the primary somatosensory cortex, specifically over the upper, lower and trunk domain areas. In vivo 2PLSM was performed using the same microscope and software as previously described (Tang et al., 2021) and was performed during the mouse light cycle. To image blood vessels, mice were intravenously (via the tail vein) administered 2% w/v fluorescein isothiocyanate (FITC)‐dextran in saline (70,000 Da; Sigma‐Aldrich, USA) 10 min prior to imaging. Mice were anesthetised with isoflurane in a chamber, then transferred to a stereotaxic frame, where isoflurane was delivered through a facemask at 2%–3% concentration in oxygen, as required, to maintain anesthesia (Tang et al., 2021). An EC Plan‐Neofluar 20X/0.1 water immersion objective (Nikon, USA), illuminated by white light, was used to identify large blood vessels in the somatosensory cortex (layers II/III) that were subsequently used as landmarks for regions of interest (ROIs). The resultant brightfield image was captured using XCAP Image Processing Software (EPIX Incorporated, USA). Once ROIs were identified for imaging, z‐plane coordinates were saved. The same z‐plane coordinate was initiated using the 2PLSM, and a higher‐resolution ROI captured. Femto second‐pulsed infrared excitation was from a mode‐locked Ti‐sapphire laser tuned to 910 nm and equipped with group velocity dispersion compensation (Mai Tai DeepSee; Spectra‐Physics, Australia). Power delivered to the back aperture was 40–100 mW (14%–17% power), depending on depth. This approach allowed the visualization of NG2DsRed, CX_3_CR1^+/GFP^ and FITC‐dextran signals, simultaneously. All ROIs were imaged with a zoom of 2.0 (210 μm × 210 μm field of view) with 1 μm z‐step intervals, beginning from a depth of 20 μm below the brain's surface down to 130 μm.

### 2.5 Processing and labeling of tissue from a human Alzheimer's disease cohort

#### 2.5.1 Human cohort details

Research using human tissue was approved by the University of Tasmania's Human Medical Research Ethics Committee (H20078) in accordance with the NHMRC National Statement on Ethical Conduct in Human Research (2018). Human brain tissue was obtained from the Banner Sun Health Research Institute's Brain and Body Donation Program. All subjects, or their legal representatives, signed an Institutional Review Board‐approved informed consent form for brain donation (for more information, see Beach et al., 2015). Briefly, human brain tissue was fixed with 4% formaldehyde, cryoprotected in 2% dimethyl sulfoxide/20% glycerol, sectioned at 40 μm on a sledge‐type microtome and free‐floating sections were stored in PBS with 0.02% sodium azide at 4°C.

Sections from the superior frontal gyrus (SFG) of 12 control cases and 11 AD cases were used in the current study (summarized in Table 1). Cases were considered to have AD if, at a minimum, they were defined as intermediate or high on the NIA‐Reagan criteria (Hyman et al., 2012). These pathological assessments were made in the frontal, temporal and parietal lobes, hippocampal CA1 region and entorhinal/transentorhinal region and pathology was present in all these regions in the AD cases (Beach et al., 2015; National Institute on Aging and Reagan Institute Working Group on Diagnostic Criteria for the Neuropathological Assessment of Alzheimer's Disease (1997)). A case was considered a control if the person did not have dementia or parkinsonism during life and were without any major neuropathological diagnosis. The SFG was selected for the current analysis as it is an area susceptible to the development of amyloid plaques and tau tangles in AD (Insel et al., 2020; Kocagoncu et al., 2020). See Supplementary Table 1 for extended detailed pathological information about the individual cases included in this study including plaque density, Cerad NP scores, Braak scores and NIA‐R designations.

**TABLE 1:** Summary demographics of human brain tissue cases.

Demographics	Control (*n* = 12)	AD (*n* = 11)
Number of cases by sex: male/female (percentage of cases)	5 (42%)/7 (58%)	5 (45%)/6 (55%)
Mean age at death: years (range)	83.8 (53–95)	80.2 (61–89)
Mean post‐mortem interval: hours (range)	2.88 (2–3.95)	2.73 (1.75–5)
Number of cases with each APOE genotype		
ɛ2/ɛ2	1	‐
ɛ3/ɛ3	10	5
ɛ3/ɛ4	‐	6
ɛ4/ɛ4	1	‐

#### 2.5.2 Labeling and imaging of pericytes, microglia and blood vessels in human brain sections

Free‐floating human SFG sections were permeabilised in PBS with 0.3% Triton X‐100 for 1 h at RT, incubated in citric acid antigen retrieval buffer (pH 4.5) overnight at 4°C, and then microwaved (650 W, 30 s) in 10 mL of fresh citric acid antigen retrieval buffer. Sections were cooled to RT for 30 min, washed in PBS with 0.3% Triton X‐100 (3 × 5 min), and incubated in serum free blocking solution (Dako, Cat# X090930‐2) for 1 h at RT. Sections were incubated at 4°C for three nights with rabbit‐anti‐PDGFRβ (1:100; Thermofisher, Cat# ab32570, RRID: AB_777165), guinea pig‐anti‐IBA1 (1:500; Synaptic Systems, Cat# 234004, RRID: AB_2493179) and UEA‐1 DyLight^TM^ 594 (1:1000; Vector Laboratories, Cat# DL‐1067), in antibody diluent (Dako, Cat #S0809). Sections were rinsed in PBS with 0.3% Triton X‐100 (3 × 10 min) and then incubated with goat‐anti‐guinea pig 647 (1:1000; ThermoFisher Cat# A21450, RRID: AB_2735091) and donkey‐anti‐rabbit 488 (1:1000; ThermoFisher Cat# A21206, RRID: AB_2535792) overnight at 4°C. Sections were washed in PBS with 0.3% Triton X‐100 (3 × 10 min), incubated in PBS containing DAPI (1:5000, Invitrogen, Cat# D3571, RRID: AB_2307445, 1 × 10 min), slide mounted (Dako, Cat# K802021‐2), briefly air dried and incubated in Trueblack, according to the manufacturer's instructions (Biotium, Cat# 23007). Sections were rinsed with distilled water and cover slipped with fluorescent mounting medium (Fischer Scientific, Cat# TA‐030‐FM). Human tissue was imaged with both a VS120 Virtual Slide System and confocal microscope using the same protocols described above for IB4 stained NG2DsRed × CX_3_CR1^+/GFP^ mouse brain sections. The researcher performing immunohistochemistry was blinded to case type throughout experimental procedures and analysis.

### 2.6 Image analysis: NG2DsRed × CX_3_CR1 ^+/GFP or GFP/GFP^ mouse brains

#### 2.6.1 Quantification of CAM and PEM prevalence in the somatosensory cortex

CAM and PEM prevalence was manually quantified in IB4 labeled NG2DsRed × CX_3_CR1^+/GFP^ tissue using QuPath 0.3.2 (Bankhead et al., 2017). Boxed ROIs (800 × 600 μm) were placed in the somatosensory cortex of both hemispheres. All “in focus” microglia and pericytes containing a DAPI‐positive nucleus were counted. Cells were not counted if their nuclei touched the edge of the box. NG2DsRed‐positive pericytes were only counted if they were associated with a vessel (specifically vessels <10 μm in diameter). Microglia were counted as a CAM if the center of their nuclei was <10 μm from the center of the closest IB4‐positive blood vessel (specifically vessels <10 μm in diameter), similar to previous approaches (Bisht et al., 2021; Mondo et al., 2020). Microglia were counted as PEM if the center point of the microglia nucleus was <10 μm from the center point of a pericyte nucleus (specifically pericytes on vessels <10 μm in diameter). They were counted as PEM irrespective of whether they had also been designated a CAM and vice‐versa. To determine if microglia and pericyte associations occur more than expected by chance, Monte‐Carlo simulations were performed, as described in Supplementary Methods.

Microglia, pericytes, CAM and PEM were also analyzed in IB4‐stained brain sections from NG2DsRed × CX_3_CR1^GFP/GFP^ mice and compared to a different cohort of 12‐week‐old NG2DsRed × CX_3_CR1^+/GFP^ mice using the same protocol above, with modification. The same somatosensory regions were traced and, using the “create tiles” function on QuPath, a 400 × 400 μm grid was placed within the traced areas. Microglia and pericytes were counted in the first complete 400 × 400 μm square, with every second complete square thereafter counted to avoid sampling bias, until six full boxes per hemisphere were counted (12 counted in total per brain). Any incomplete squares or squares containing artifacts (e.g., tissue rips, folds or other artifacts that affected cell counting) were excluded. Cells contacting the edges of the squares were only counted if their nuclei were touching the top, or left‐hand margins of the box, to avoid double counting. The researcher performing the analysis was blinded to genotype throughout quantification.

#### 2.6.2 Quantifying vessel width beneath CAM, PEM and pericytes along capillaries

Capillary width at CAM and PEM was quantified in NG2DsRed × CX_3_CR1^+/GFP^ and NG2DsRed × CX_3_CR1^GFP/GFP^ tissue using QuPath 4.2. All measurements were made on vessel segments without branchpoints. For CAM, a single line was drawn (using the line tool) across the IB4‐positive basement membrane, from the center of the microglia cell body. For the purposes of this analysis, a measurement was only made at CAM that were >10 μm from a pericyte, to avoid including PEM. For PEM, lines were drawn across the basement membrane on either side of the pericyte cell body and the average of these two lines was used to determine vessel width. Measurements were made on vessels <10 μm in diameter and were conducted by a researcher blinded to genotype.

#### 2.6.3 Quantifying AQP4 coverage of pericytes with or without a PEM

AQP4 coverage of pericytes with or without an associated PEM was quantified from 100× confocal images using FIJI‐ImageJ. Exposure times for AQP4 imaging and z‐stacks (1 μm) were kept consistent for all images. Eight 1 μm planes were segregated from the larger z‐stacks based on the planes in which pericytes were in focus. Pericytes were ~5–6 μm in diameter, so eight planes ensured AQP4 coverage was assessed across entire pericyte cell bodies. These planes were z‐stacked using max projection, and pericyte cell bodies were traced on each image to create ROIs. These ROIs were expanded by 0.25 μm in all directions to ensure AQP4 labeling was assessed in the area overlaying the pericyte. To prevent bias, pericytes with or without a PEM were traced with the CX3CR1 channel turned off. The same eight focal planes were then segregated in the AQP4 images and were z‐stacked using sum‐slices. Background was subtracted from these z‐projections using the subtract background function with a rolling ball radius of 50 pixels. The previously traced ROIs were applied to the stacked AQP4 images, and the mean gray value was measured within these ROIs, to determine the average pixel intensity of AQP4 labeling surrounding each pericyte.

### 2.7 Image analysis: in vivo NG2DsRed × CX_3_CR1 ^+/GFP^ two‐photon derived images

#### 2.7.1 Quantification of CAM and PEM

Using FIJI‐ImageJ (NIH, USA) (Schindelin et al., 2012), images were automatically adjusted for brightness/contrast. The plugin “Cell Counter” was used to manually annotate the pericytes and microglia in each ROI. Microglial GFP signal and FITC‐dextran vessel lumen signal was differentiated by the higher intensity of the GFP compared to the FITC and the presence of dark lines indicating red blood cells within the vessel lumen. The distances between pericytes and microglia, and microglia and vessels, were calculated based on x, y, and z co‐ordinates for the center point of each cell soma, or vessel lumen, using FIJI‐ImageJ. The same distances were used to define PEM and CAM as described in Section 2.6.1. Microglia were counted as PEM irrespective of whether they had also been designated a CAM and vice‐versa.

#### 2.7.2 Quantification of pericyte, CAM and PEM vascular tree location

Using images obtained on imaging day −1 (with FITC‐dextran, see Figure 2), the position of vessels within the vascular tree was able to be determined from z‐stacks of 10 ROIs in a subset of five mice, as previously illustrated (Hall et al., 2014). Penetrating arterioles (0 order) were identified by their large luminal size and presence of NG2DsRed‐positive rings of vascular smooth muscle cells (VSMCs). Branching higher order capillaries (1st–2nd order) were identified by their smaller vessel width and presence of transitional ensheathing pericytes. Mid‐order (3rd–4th) and lower order capillaries (≥5th order) were identified by their small vessel width and presence of thin‐strand pericytes. Ascending venules (8th order) were identified by their larger luminal size and mesh pericyte processes with a distinct lack of ringed VSMCs. See Figure 2 for an example ROI encompassing an entire vascular tree. The position of pericytes, CAM and PEM were then quantified at different levels of the vascular tree.

#### 2.7.3 Tracking microglia proximity to pericytes over time

The *x*, *y*, and *z* co‐ordinates of all microglia and pericytes within a ROI was used to determine the relative distance between the cells on each day of imaging. On day 0, 32 pericytes from six mice were identified to have a PEM. These 32 pericytes with a PEM on day 0, were re‐imaged on days 4, 7 and 28 to determine if microglial associations with pericytes are stable or dynamic. The total number of pericytes and pericytes with a PEM in each ROI was also quantified on each imaging day, to calculate the percentage of pericytes with a PEM on days 0, 4, 7 and 28.

#### 2.7.4 Determining vessel width at locations of CAM, PEM, and pericytes along capillaries

Using the line tool in FIJI‐ImageJ, images taken on day −1 from the six mice tracked longitudinally (when FITC‐dextran was administered to visualize vessels), plus two additional mice, were manually annotated to measure vessel width of capillaries at four specific locations: vessel only (VO), containing no pericyte or microglia cell bodies; pericyte on vessel (P), where a pericyte was present alone on a vessel; CAM, where a microglia alone was associated with a vessel; and PEM, where a microglia was associated with a pericyte on a vessel (Figure 5f). All measurements were made on vessel segments without branchpoints. For VO, a line was drawn across the vessel. For pericytes, CAM and PEM, a line was drawn across the vessel at the center of the pericyte (for pericytes and PEM) or microglia (for CAM) cell bodies. Images taken on day −1 and day 28 in the six mice tracked longitudinally (when FITC‐dextran was administered to visualize vessels) were also manually annotated to measure vessel width at locations where a pericyte identified on day −1 had either gained or lost a PEM by day 28. Vessel measurements were only made from biological replicates in which the FITC‐dextran signal was strong enough for quantification. For the purposes of this analysis, width at CAM was only quantified if the CAM was >10 μm from a pericyte, to avoid including PEM. Measurements were made on vessels <10 μm in diameter.

### 2.8 Analysis of PEM using a publicly available 3D electron microscopy data set

A number of 3D electron microscopy (EM) data sets from the brains of various species have recently become available (see Bonney et al. [2022] for more information). We probed the Cortical MM^3 data set (Bae et al., 2021), generated by the Machine Intelligence from Cortical networks (MICrONS) consortium, to find examples of PEM in mouse brain tissue. This data set is derived from serial section transmission EM images that span 1.3 × 0.87 × 0.82 mm^3^ of a postnatal day 87 SLC17a7‐Cre::Ai162 male mouse visual cortex. Microglia were identified by their heterochromatin pattern and elongated, irregular shape (Peters & Folger, 2013; Savage et al., 2018). We also commonly observed inclusions within the perikarya, which are likely phagocytosed material (Savage et al., 2018). Pericytes were identified as being enclosed by the basement membrane, by their position on the abluminal surface of endothelial cells and by their protruding cell bodies, as previously described (Alarcon‐Martinez et al., 2021; Bonney et al., 2022). Astrocytes are less electron dense, so their processes were identifiable by their lighter appearance compared to neighboring cells (Bonney et al., 2022). One downside of this data set is that the segmentation is not always accurate, so some separate cellular elements become pseudo colored together in the 2D images and 3D reconstructions. We provide the raw greyscale EM images and coordinates of the interacting cells in the figure legend. The data set can be accessed online at https://www.microns-explorer.org/cortical-mm3.

### 2.9 Image analysis: Pericytes, microglia, CAM and PEM in human brain sections

Pericytes and microglia were manually counted using QuPath 3.0. ROIs were created by tracing the entire gray matter in each section, and then shrinking the trace by 50 μm to remove edge artifacts. Other obvious artifacts (e.g., tissue rips, folds, etc.) were manually removed from the ROIs. Using the “create tiles” function on QuPath, a 500 × 500 μm grid was placed within each ROI. Microglia and pericytes were counted in the first complete 500 × 500 μm square within the ROI, with every fourth complete square counted thereafter to avoid sampling bias, until 20 full squares were counted. Any incomplete squares or squares containing artifacts (e.g., tissue rips, folds or other artifacts that affected cell counting) were skipped. Cells contacting the edges of the squares were only counted if their nuclei were touching the top, or left‐hand margins of the box, to avoid double counting. Cells were only counted if there was a clear DAPI‐positive nucleus present. Pericytes were identified based on PDGFRβ‐positive labeling and morphology. Specifically, in human tissue PDGFRβ labeled both the processes and cell body of pericytes in a punctate pattern, with enhanced labeling around the cell body. Pericytes were also distinguished from other cells on vessels (i.e., endothelial cells) by their bump‐on‐log morphology. Microglia were identified by labeling with IBA1 and by their distinct ramified morphology. Examples of these are provided in Supplementary Figure 1. CAM and PEM were identified using the approach described for mouse tissue above (Section 2.6.1), using UEA‐1 labeling to identify capillaries. The researcher performing the analysis was blinded to genotype throughout quantification.

### 2.10 Statistical analysis

Data were processed in Microsoft Excel and all statistical analysis was performed using GraphPad Prism 9.3.1 (GraphPad, USA). Prior to statistical analysis, data underwent outlier testing using a ROUT test (*Q* = 1%) and outliers were removed from data sets unless they were biologically relevant. Data were tested for normality of the residuals using the D'Agostino & Pearson test, or the Shapiro–Wilk test if *n* numbers were too small for the D'Agostino & Pearson. For unpaired two group comparisons, means were compared using an unpaired *t*‐test if data were normally distributed, but a Welch's correction was applied if variance was significantly different as tested by an *F* test. If data were not normally distributed in any group, a Mann–Whitney test was employed. For paired two group comparisons, a paired *t*‐test was applied if data were normally distributed, and a Wilcoxon test applied if data were not normally distributed in any group. For comparisons with more than two groups, if data were normally distributed a repeated measures one‐way ANOVA with a Geisser–Greenhouse correction was applied to account for variations in sphericity and Tukey's post‐hoc test employed for multiple comparisons. If repeated measures data were not normally distributed, a Friedman test followed by a Dunn's post‐hoc test was applied. A *p* < .05 was considered statistically significant. Statistical tests used for each analysis are reported in figure legends.

## 3 RESULTS

### 3.1 A subset of microglia are adjacent to capillary pericytes in the healthy mouse brain and spinal cord

**FIGURE 1:**
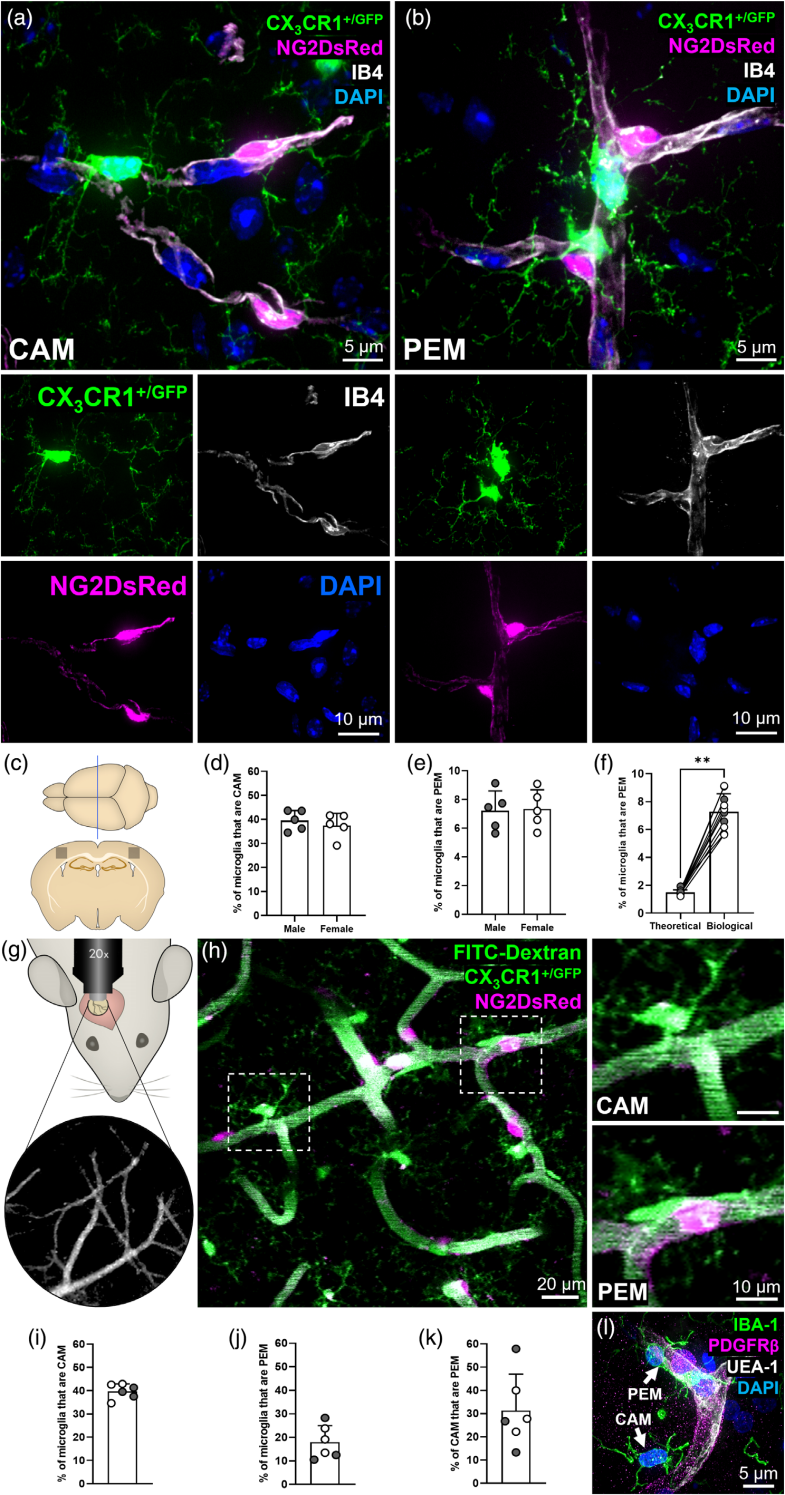
A subset of microglia are directly adjacent to pericytes. (a) Representative example of a capillary‐associated microglia (CAM), in the somatosensory cortex, extending fine processes to contact other DAPI‐positive nuclei. (b) Representative example of two pericyte‐associated microglia (PEM), in the hippocampus, with the bottom PEM morphologically curved around a pericyte. (a, b) For all images: NG2DsRed‐positive pericytes (magenta), CX_3_CR1^+/GFP^‐positive microglia (green), IB4‐labeled vessels (white) and DAPI‐labeled nuclei (blue). Each fluorescent channel alone is located below the main image. All images were derived from 12‐week‐old NG2DsRed × CX_3_CR1^+/GFP^ mice using confocal microscopy. (c) Schematic of region analyzed within the somatosensory cortex of NG2DsRed × CX_3_CR1^+/GFP^ mice (Bregma −1.5 mm [Allen Reference Atlas, n.d.]). (d, e) Quantification of (d) CAM and (e) PEM in the somatosensory cortex of male (*n* = 5) and female (*n* = 5) mice. Data compared with an unpaired parametric *t*‐test. (f) The predicted percentage of PEM when running simulations with microglia and pericyte densities derived from 10 different biological replicates, compared to the actual PEM percentage from those same 10 somatosensory biological replicates (biological data derived from [e]). Data compared with a Wilcoxon test. Lines represent paired data. (g) Schematic of cranial window location in NG2DsRed × CX_3_CR1^+/GFP^ mice with blood vessels used as landmarks. (h) Representative 30 μm thick projection image of NG2DsRed‐positive pericytes (magenta), CX_3_CR1^+/GFP^‐positive microglia (green) and FITC‐dextran‐positive vessel lumen (green) in layers II/III of the somatosensory cortex of adult NG2DsRed × CX_3_CR1^+/GFP^ mice imaged using 2PLSM. Dashed boxes highlighting a CAM and PEM are magnified in panels to the right. (i–k) Quantification of the percentage of microglia that are (i) CAM, (j) PEM and (k) the percentage of CAM that are PEM, in layers II/III of the somatosensory cortex of male (*n* = 3) and female (*n* = 3) mice. (l) Representative image of PDGFRβ‐positive pericyte (magenta), IBA1‐positive microglia (green), UEA‐1‐labeled vessels (white) and DAPI‐labeled nuclei (blue) from the SFG of a human control brain (93 y.o. female). A CAM and PEM are highlighted by white arrows. For all graphs, gray circles represent males and white circles represent females. Data presented as mean ± SD. **p < .01.

Given there has not yet been a formal investigation of the spatial relationship between microglia and pericytes, we first determined if microglia and pericytes reside adjacent to one another and how often such associations occur in the healthy brain. To do this, we generated NG2DsRed × CX_3_CR1^+/GFP^ mice to enable robust visualization of microglia (CX_3_CR1^+/GFP^) and pericytes (NG2DsRed) in fixed brain tissue (Figure 1a,b). We note that the NG2DsRed line has previously been used to visualize oligodendrocyte progenitor cells (OPCs (Hartmann et al., 2015; Nemes‐Baran et al., 2020), Supplementary Figure 2), and that some OPCs have been found to reside on the vasculature (Pfeiffer, 2022; Pfeiffer et al., 2021). It is therefore possible OPCs could be mistaken for pericytes when using this genetic line.

To determine how often OPCs are NG2DsRed‐positive in this line compared with pericytes, we labeled tissue derived from NG2DsRed mice with PDGFRβ (a marker of capillary pericytes) or PDGFRα (a marker of OPCs). Only 7.5 ± 0.9% of NG2DsRed‐positive cells were co‐positive for PDGFRα (Supplementary Figure 2F). In contrast, when NG2DsRed‐positive cells were labeled with PDGFRβ, 93.5 ± 5.3% were co‐positive (Supplementary Figure 2G). This suggests the vast majority of NG2DsRed‐positive cells are capillary pericytes, not OPCs. To understand why so few NG2DsRed cells were co‐positive for PDGFRα, we next quantified the total number of PDGFRα cells co‐positive for NG2DsRed. We found that only 12.5 ± 4.1% of PDGFRα‐positive OPCs were co‐positive for NG2DsRed (Supplementary Figure 2H), suggesting many OPCs do not express detectable levels of DsRed in the NG2DsRed mouse line. In contrast, the vast majority of PDGFRβ‐positive capillary pericytes were dual‐positive for NG2DsRed (98.9 ± 0.6%, Supplementary Figure 2I). Those PDGFRα‐positive OPCs that were dual‐positive for NG2DsRed also exhibited a weaker DsRed signal than pericytes positive for NG2DsRed (Supplementary Figure 2J). Combined, this data suggested that if we were to quantify NG2DsRed‐positive cells on the vasculature using this line without regard for morphology, the vast majority would be pericytes, not OPCs. In addition, throughout this study we used manual quantification and verification. This approach enabled us to consider both pericyte and OPC morphology while quantifying, allowing us to select cells exhibiting pericyte morphology that were on the vasculature and to avoid NG2DsRed‐positive OPCs with clear OPC morphological features, even when they were associated with vessels.

Confocal microscopy of NG2DsRed × CX_3_CR1^+/GFP^ brain tissue sections highlighted a population of microglia that closely associates with capillaries, which we refer to as capillary‐associated microglia (CAM, Figure 1a, Supplementary Movie S1), consistent with a previous report (Bisht et al., 2021). We then observed a second newly identified population of microglia that overlap with CAM, but which are located directly adjacent to pericytes on capillaries (Figure 1b, Supplementary Figure 3, Supplementary Movies S2 and S3). We have termed these pericyte‐associated microglia (PEM). Both CAM and PEM were observed extending processes toward other nuclei, suggesting they may make multiple contacts with blood vessels, pericytes and other cell soma in the brain parenchyma simultaneously (Figure 1a,b, Supplementary Figure 3, Supplementary Movies [Link], [Link]). We also frequently observed PEM soma exhibiting a curved morphology around pericyte cell bodies (Figure 1b,l, Supplementary Figure 3, Supplementary Movies S2 and S3, Figure 3b) and the processes of both PEM and CAM reaching out to enfold or contact pericyte cell bodies (Figure 1b and 3a,c).

Quantification revealed a large proportion of microglia were CAM (male = 39.6 ± 4.2%, female = 37.5 ± 5.1%, Figure 1c,d), which is consistent with previous reports (Bisht et al., 2021). Next, we assessed the proportion of microglia that were PEM, finding in tissue slices ~1 in 15 microglia were located adjacent to pericytes (male = 7.2 ± 1.4%, female = 7.3 ± 1.3%, Figure 1c,e). There were no significant differences in the proportion of microglia that were CAM or PEM between male and female mice (Figure 1d,e). To determine whether the proportion of microglia that were PEM was higher than would be expected by random chance, we ran simulations to mathematically model chance associations between pericytes and microglia. We found the percentage of PEM to be ~5 fold more than expected by chance (7.3 ± 1.3% vs. 1.5 ± 0.19%, Figure 1f, Supplementary Figure 4).

To determine if PEM are a ubiquitous feature of the CNS, we expanded our analysis of microglia‐pericyte associations to also include the caudate putamen, hippocampus, thalamus, hypothalamus and the spinal cord (Supplementary Figure 5). The proportion of microglia identified as PEM was consistent across all regions assessed (Supplementary Figure 5B–D), though there were some differences in the number of pericytes and microglia and the proportion of pericytes with a PEM (Supplementary Figure 5E–G). Furthermore, we identified the presence of both CAM and PEM in the thoracic region of the spinal cord of NG2DsRed × CX_3_CR1^+/GFP^ mice (Supplementary Figure 5H–L).

We next replicated and extended this analysis by quantifying the proportion of microglia that were CAM and PEM in the upper layers of the somatosensory cortex (layers II/III), using images derived from in vivo 2PLSM taken through cranial windows (Figure 1g). This approach allowed us to assess the spatial relationship between pericytes and microglia in three dimensions through large z‐stacks (~130 μm in depth, Figure 1h, Supplementary Movie S4). Similar to our analysis in fixed tissue, we found that ~2 in every 5 microglia were CAM (39.7 ± 3.2%, Figure 1i), but that ~1 in every 6 microglia were PEM (18.0 ± 7.0%, Figure 1j), a higher proportion than in our fixed tissue analysis. Regions were manually selected for in vivo imaging if they were specifically enriched in PEM, likely partially explaining this difference. Of the microglia that were CAM, one third of them also met our definition of PEM (31.3 ± 15.7%, Figure 1k). We also identified both PEM and CAM in the SFG of post‐mortem human tissue (Figure 1l, Supplementary Movie S5), illustrating they are conserved across species.

**FIGURE 2:**
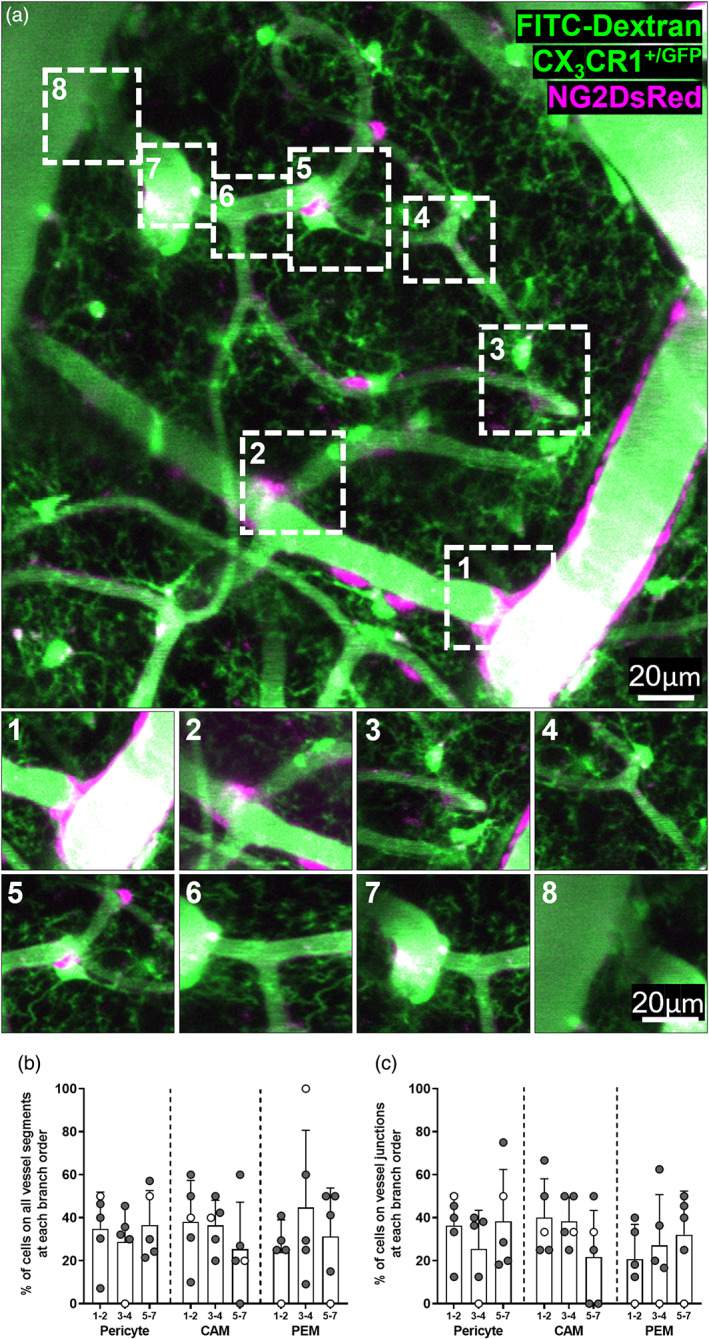
CAM, PEM and pericytes are present at all branch orders of capillaries. (a) Representative image stack (20–75 μm depth, average intensity projection) of FITC dextran filled vessels in the somatosensory cortex of an NG2DsRed × CX_3_CR1^+/GFP^ mouse imaged in vivo using 2PLSM. Pericytes are labeled magenta, and microglia and vessels are green. Insets correspond to boxes labeled 1–8 in main panel. (1) Penetrating arteriole (0th order) branching off to form a capillary (first order). (2–7) Higher order capillaries branching. (8) Seventh order capillary converging on the ascending venule. (b, c) Percentage of total CAM, PEM and pericytes that are located (b) at different branch orders of the vascular tree, and (c) at vessel junctions at different branch orders of the vascular tree (*n* = 5, four male and one female). For all graphs, gray circles represent males and white circles represent females. Data presented as mean ± SD.

Given that pericytes reside throughout the entire capillary bed, we used the in vivo 2PLSM images to determine if CAM and PEM preferentially reside at specific levels of the vascular tree (Figure 2, Supplementary Figure 4). We observed CAM, PEM and pericytes at every level of the vascular tree (Figure 2b), at both vessel junctions (Figure 2c) and straight vessel segments (Supplementary Figure 4C), with no clear positional preference. Collectively, these findings illustrate that a subset of microglia are directly adjacent to pericytes in the mouse brain and spinal cord, as well as the human brain and that they are evenly distributed throughout the vascular tree.

### 3.2 Microglia can directly associate with the basement membrane covering pericytes in between astrocyte endfeet

**FIGURE 3:**
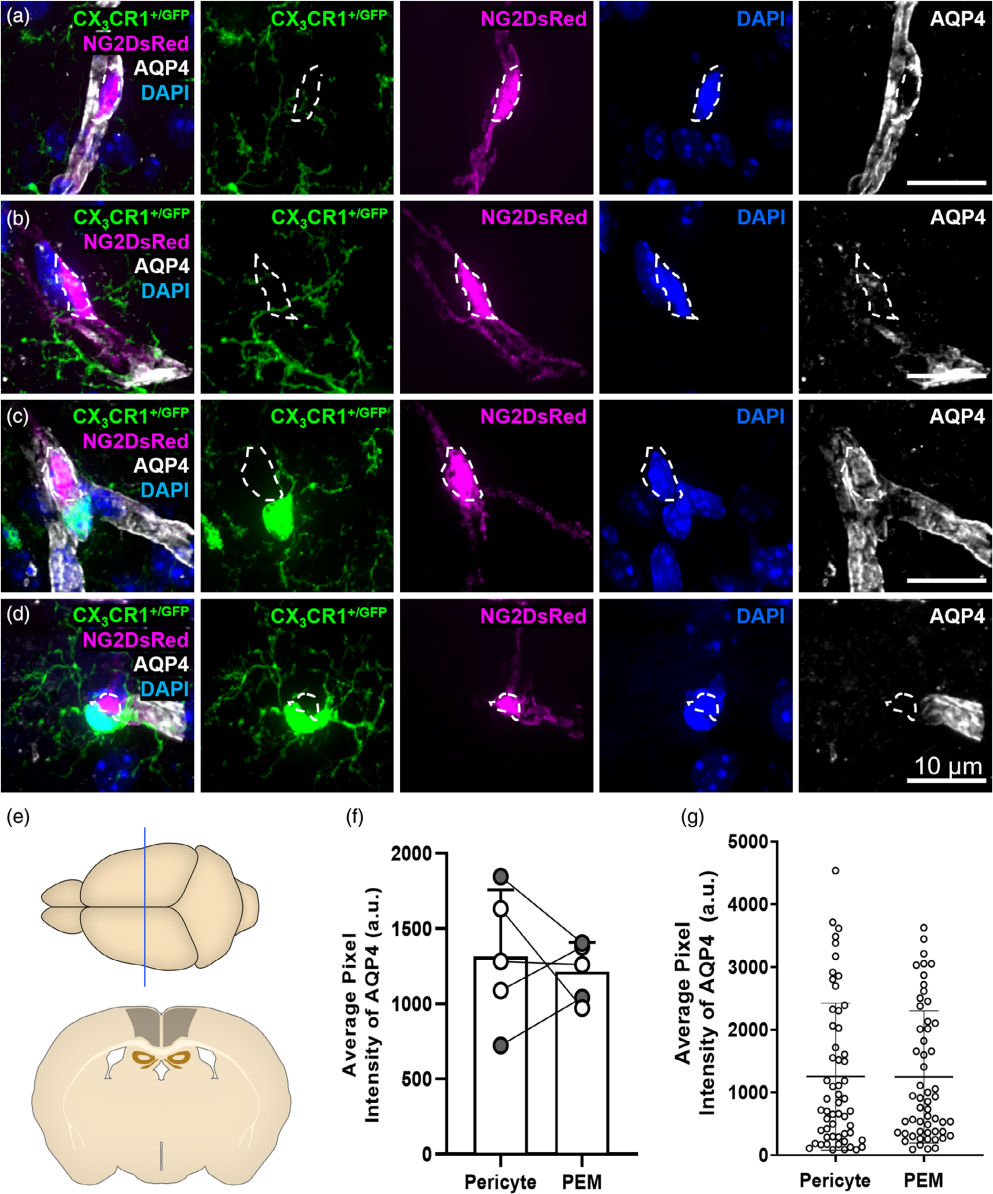
Microglia can interact with pericytes with or without AQP4‐positive astrocyte endfeet. (a) Representative example of a pericyte (magenta) with no associated PEM exhibiting strong labeling by AQP4 (white). (b) Representative example of a pericyte (magenta) that is not associated with a PEM, but lacking AQP4 (white) labeling. (c) Representative example of a pericyte (magenta) associated with a PEM (green). The pericyte is exhibiting strong labeling by AQP4 (white). (d) Representative example of a pericyte (magenta) associated with a PEM (green). The pericyte is lacking AQP4 (white) labeling. (e) Schematic of region analyzed in 12‐week‐old NG2DsRed × CX_3_CR1^+/GFP^ mice (~Bregma −1.10 mm [Allen Reference Atlas, n.d.]). (f) Quantification of AQP4 fluorescence intensity in regions overlaying pericytes with, or without, an associated PEM (*n* = 5, two males, three females). Data compared with a paired parametric *t*‐test. (g) AQP4 fluorescence intensity around individual pericytes with, or without, an associated PEM (*n* = 5, two males, three females). This data was used to derive the averages in (f). Data are presented as mean ± SD. For all images: NG2DsRed‐positive pericytes (magenta), CX_3_CR1^+/GFP^‐positive microglia (green), AQP4‐labeled astrocyte endfeet (white) and DAPI‐labeled nuclei (blue) are shown. Images showing each fluorescent channel alone are to the right of the main image.

It has previously been demonstrated that microglia can associate with vessels without the interference of astrocyte endfeet coverage (Bisht et al., 2021; Mondo et al., 2020). To test whether pericytes with an associated PEM were less likely to be covered by astrocyte endfeet compared to pericytes without a PEM, we labeled 12‐week‐old NG2DsRed × CX_3_CR1^+/GFP^ tissue with AQP4, a marker of astrocyte endfeet. We found examples of pericytes both covered by AQP4 and devoid of AQP4 labeling, irrespective of whether they had an associated PEM (Figure 3a–d). Confocal images (Figure 3d, Supplementary Figure 7), 3D reconstructions (Supplementary Movies [Link], [Link]) and depth shading (Supplementary Figure 7C) illustrated that microglial cell bodies and processes can associate with pericytes lacking AQP4 labeling. When we quantified the fluorescence intensity of AQP4 around pericytes, we found no difference in AQP4 coverage of pericytes with or without a PEM, suggesting that a lack of AQP4 labeling is not a unique feature of pericytes with a PEM (Figure 3e–g).

**FIGURE 4:**
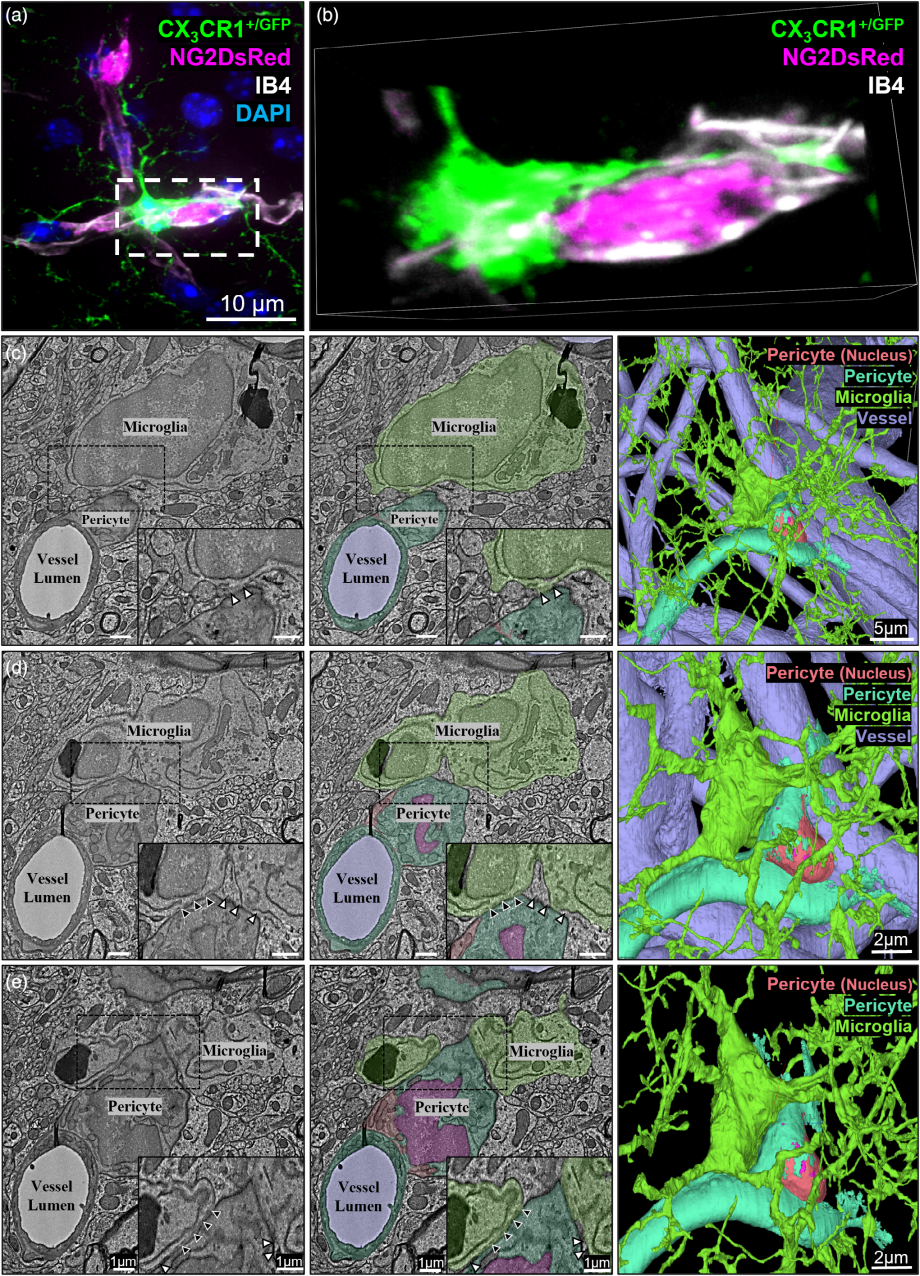
Microglia can directly associate with the basement membrane covering pericytes. (a) Representative example of a pericyte‐associated microglia extending a process around a pericyte at the border of the somatosensory and motor cortex (~Bregma −1.5 mm). This representative image shows the basement membrane between a microglial cell body and process and a pericyte soma. NG2DsRed‐positive pericyte (magenta), CX_3_CR1^+/GFP^‐positive microglia (green), IB4‐labeled vessels (white) and DAPI‐labeled nuclei (blue). (b) Magnification of dashed box in (a), rotated to illustrate the presence of the basement membrane (IB4, white) between the microglia (green) and pericyte (magenta). All images were derived from 12‐week‐old NG2DsRed × CX_3_CR1^+/GFP^ mice using confocal microscopy. Individual channels are shown in Supplementary Figure 7D. (c–e) Representative serial section transmission EM images of a microglia (green) associating with a pericyte (orange, magenta and cyan) on a capillary (light‐purple), through a stack starting at a z‐depth of (c) 0 nm, (d) 800 nm and (e) 1200 nm. Left column shows raw images, center column shows cellular components pseudo colored. Dashed boxes in left and center column images are magnified in the bottom right corner of each image. The EM images in the left column show an endothelial cell (bottom left of all images) segregated from the pericyte by a basement membrane. In the center column these cells have not been segmented separately, so have been pseudo colored the same (cyan). Although we found examples of pericytes and endothelial cells correctly segmented in this dataset, it is common to find mistakes in the segmentation, as observed by others (Bonney et al., 2022). Black arrowheads indicate where the microglia cell body is in direct contact with the basement membrane covering the pericyte cell body. White arrowheads indicate where astrocyte endfeet are observable between the two cells. The right column shows 3D reconstructions of the pericyte and microglia; top right is zoomed out with all colors highlighted, second top right is a magnified view of the top right image and bottom right is with the vessels removed. Coordinates are: *X*: 365279, *Y*: 175038, *Z*: 23443. Images generated from https://www.microns‐explorer.org/cortical‐mm3.

The 3D reconstructions (Supplementary Movies [Link], [Link], Supplementary Figure 7B,C) also revealed gaps between the associating microglia and pericytes, despite the lack of AQP4 labeling between them (Supplementary Figure 7A–C, Supplementary Movies S7 and S8). Staining of the basement membrane revealed IB4‐positive basement membrane between microglia and pericytes (Figure 4a,b, Supplementary Figure 7D,E, Supplementary Movies S9 and S10). Together, these findings illustrate that PEM likely reside outside of the basement membrane but can reside inside the AQP4‐positive astrocyte endfeet layer, although this is not always the case.

An absence of AQP4 labeling does not necessarily mean astrocyte endfeet are absent. Therefore, to further investigate the ultrastructural relationship between pericytes and PEM, we probed the publicly available Cortical MM^3 EM data set (Bae et al., 2021) to find evidence of microglia‐pericyte interactions. We identified several microglia cell bodies within 10 μm of pericyte cell bodies. Figure 4c–e shows one example of a microglia cell body contacting the basement membrane covering a pericyte, without the interference of astrocyte endfeet, supporting the findings from our confocal imaging. The points at which microglia contact the basement membrane of pericytes likely occur through gaps in the astrocyte endfeet, as endfeet were visibly covering the pericyte outside of the points of contact (Figure 4c–e).

### 3.3 Microglial associations with pericytes can be gained and lost

**FIGURE 5:**
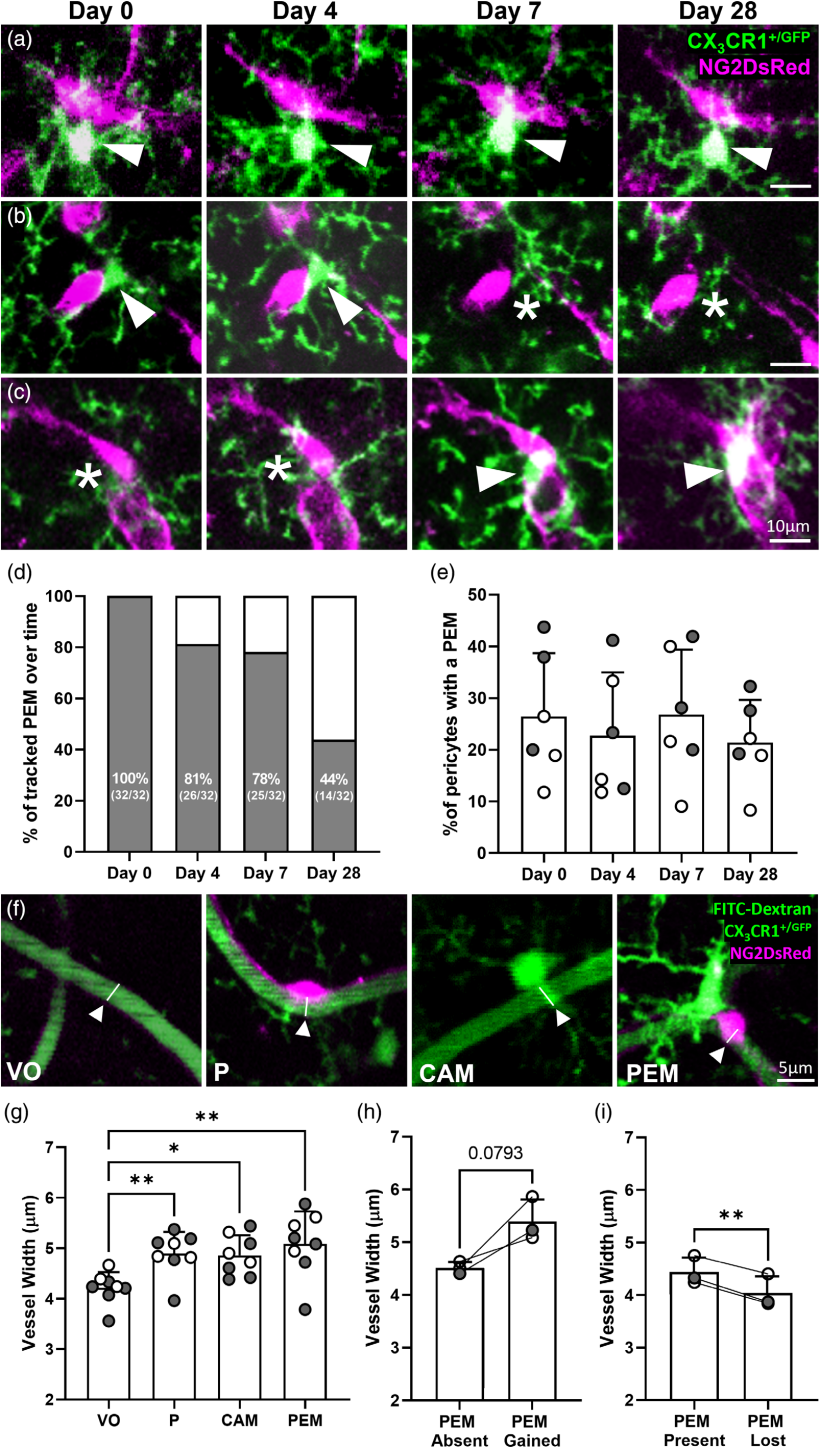
Pericytes can gain or lose PEM over 28 days and vessel width is increased beneath pericyte and microglia soma. (a) Representative in vivo 2PLSM images of a PEM that maintains position for 28 days in the somatosensory cortex of an NG2DsRed × CX_3_CR1^+/GFP^ mouse. (b) Representative images of a PEM in position for 4 days that is no longer in place on day 7 or 28 of imaging. (c) Representative images of a pericyte with no adjacent PEM on day 0 or 4 but gains a PEM on day 7. (a–c) For all images pericytes are magenta, microglia are green, and images are average intensity projections. White arrowheads highlight a PEM and white asterisks indicate the absence of a PEM. (d, e) Percentage of (d) 32 PEM, identified on day 0 of imaging, that maintain position adjacent to a pericyte on days 4, 7 and 28 of imaging and (e) total pericytes with a PEM per mouse on days 0, 4, 7 and 28 (*n* = 6, three male and three female). Data in (e) analyzed using a repeated measures one‐way ANOVA (day of imaging *F*(2.1, 10.4) = 1.8, *p* = .2137), with Tukey's post hoc test. (f) Representative images indicating locations of vessel width measurement in in vivo 2PLSM images derived from NG2DsRed × CX_3_CR1^+/GFP^ mice, following administration of the vessel lumen marker FITC‐dextran. White arrowheads indicate the midpoint below the cell soma. (g) Quantification of vessel width (μm) of vessel only (VO), and vessels with a pericyte (P), CAM or a pericyte with a PEM (150, 124, 90 and 124 vessels were measured, respectively, across *n* = 8 mice, five male and three female). Data presented as average per animal with a minimum of four vessel measurements at VO, P, CAM and PEM per animal. Comparisons were made with a Friedman's test (Friedman statistic = 14.85, *p* = .0019), with Dunn's post hoc test. (h–i) Quantification of vessel width (μm) of vessels with a pericyte where a PEM was (h) gained or (i) lost (*n* = 3 mice, 1 male and 2 female). Data presented as average per animal with a minimum of two vessel measurements. Comparisons made with a paired parametric *t*‐test. For all graphs, gray circles represent males and white circles represent females. Data presented as mean ± SD. *p < .05; **p < .01.

To determine if PEM maintain their position adjacent to pericytes over time, we tracked 32 PEM over 28 days, through cranial windows in NG2DsRed × CX_3_CR1^+/GFP^ mice (Supplementary Figure 8). Throughout the imaging period, we identified PEM that fell within three groups: PEM that remained adjacent to their partner pericytes over the entire 28‐day period (Figure 5a); PEM that were no longer adjacent to a pericyte within the 28 days (Figure 5b); and pericytes that did not originally have a PEM, but which gained one during the 28‐day period (Figure 5c). Upon quantification, we identified that the majority of PEM from day 0 were still positioned directly adjacent to a pericyte after 4 days (81%) and 7 days (78%), but by day 28, only 44% of PEM remained adjacent to a pericyte (Figure 5d). Despite this loss of PEM, we did not observe a change in the total proportion of microglia that were defined as a PEM throughout the 28 days, indicating some pericytes that did not originally have a PEM had gained a PEM during the imaging period (Figure 5e). Collectively, these data suggest microglial associations with pericytes are dynamic, are consistent in proportion over time, and only a minority of PEM have associations with pericytes that last 28 days.

### 3.4 The association of microglia and pericytes may cause alterations to vessel width beneath pericytes in the healthy adult mouse brain

To investigate if CAM or PEM may alter capillary diameter in the healthy brain, we measured capillary width beneath pericytes, CAM and pericytes with a PEM, compared to vessel segments without any observable associated pericytes, CAM or PEM (Figure 5f). We found that the average vessel width for vessels with no observable associated cells was 4.21 ± 0.32 μm (Figure 5g). Compared to vessels with no observable cells (vessel only, VO), the vessel width was significantly increased by 16% beneath pericytes (4.89 ± 0.43 μm, *p* = .0060) and by 15% beneath CAM (4.86 ± 0.40 μm, *p* = .0402). Beneath pericytes with an adjacent PEM, the width of vessels was 21% wider (5.08 ± 0.64 μm, *p* = .0060) than vessels with no observable cells (Figure 5g). These findings suggest the presence of both pericytes and microglia on capillaries is associated with increased capillary width under basal conditions, but that the presence of a PEM near a pericyte does not alter this. To investigate if the gain or loss of a PEM altered capillary diameter in the healthy brain, we measured capillary width beneath pericytes that had gained a PEM or lost a PEM within a month of imaging (Figure 5h,i). We found that the average vessel width strongly trended toward an increase when a pericyte gained a PEM during the imaging period (5.39 ± 0.41 μm, *p* = .0793) compared to when the PEM was previously absent (4.51 ± 0.11 μm; Figure 5h). Conversely, vessel width was significantly decreased by ~10% when a pericyte lost a PEM (4.04 ± 0.32 μm, *p* = .0059) compared to when the PEM was previously present (4.44 ± 0.27 μm; Figure 5i). These findings suggest that the gain or loss of a PEM at pericytes may alter capillary width in the healthy brain.

### 3.5 CX_3_CR1 knock‐out does not alter the proportion of microglia that are CAM or PEM

**FIGURE 6:**
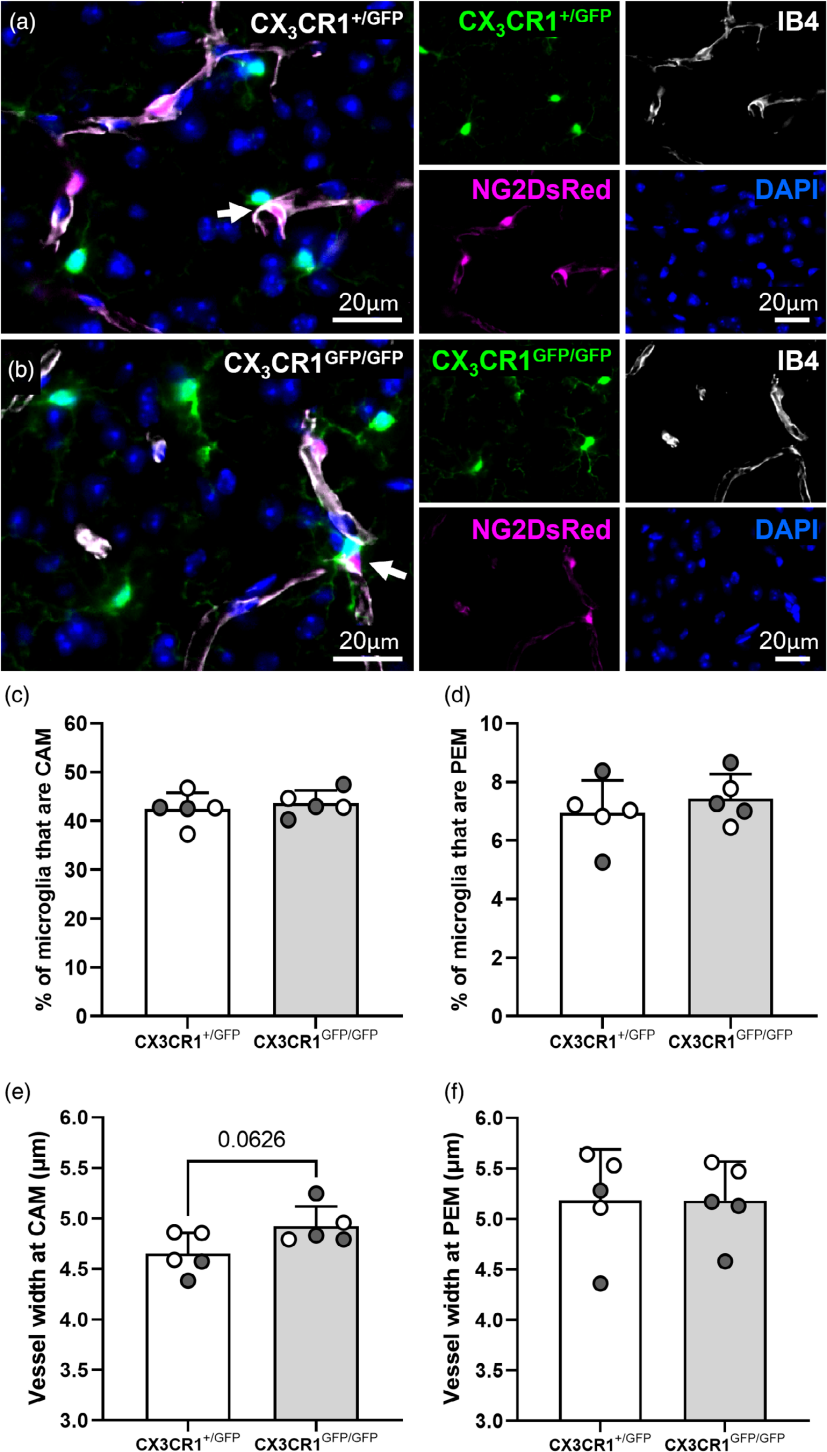
CX_3_CR1 knock‐out does not alter the proportion of microglia that are PEM or CAM and does not alter vessel width at PEM and CAM in 12‐week‐old mice. (a, b) Representative images of (a) NG2DsRed × CX_3_CR1^+/GFP^ and (b) NG2DsRed × CX_3_CR1^GFP/GFP^ mice showing that PEM are present in both mouse lines (white arrows). For each image: NG2DsRed‐positive pericytes (magenta), CX_3_CR1^+/GFP^‐positive microglia (green), IB4 (white) and DAPI‐labeled nuclei (blue) are shown. Images showing each fluorescent channel alone are to the right of the main image. (c–f) Quantification of the percentage of microglia that are (c) CAM and (d) PEM, and width (μm) of IB4 labeled capillaries at sites of (e) CAM and (f) PEM in the somatosensory cortex (c–f, Bregma −1.5; *n* = 5 per group, two males and three females for NG2DsRed × CX_3_CR1^+/GFP^, three males and two females for NG2DsRed × CX_3_CR1^GFP/GFP^). Data compared with an unpaired parametric *t*‐test. For all graphs, gray circles represent males and white circles represent females. Data presented as mean ± SD.

The signals that recruit microglia to pericytes are unknown. Microglia migrate in response to the fractalkine ligand (CX_3_CL1), which can be released from neurons and binds to the fractalkine receptor (CX_3_CR1) on microglia. Recently, CX_3_CL1 was shown to cause capillary constriction at regions where microglia contact vessels, in both the brain and retinal explants, suggesting the CX_3_CL1/ CX_3_CR1 axis may play a role in microglia modulation of vessel tone (Mills et al., 2021). Pericytes also have the capacity to release CX_3_CL1 (Smyth et al., 2018), so it is possible they may be a source of CX_3_CL1 to recruit microglia to the vasculature. To determine if microglia are recruited to vessels and pericytes through the CX_3_CL1/CX_3_CR1 axis, we took advantage of the CX_3_CR1^GFP^ knock‐in/knock‐out line, which replaces the first 390 base pairs of exon 2 of CX_3_CR1 with GFP. We derived both the heterozygous KO NG2DsRed × CX_3_CR1^+/GFP^ line and the complete knock‐out (KO) NG2DsRed × homozygous CX_3_CR1^GFP/GFP^. Homozygosity was determined through genotyping and confirmed by the observation of increased GFP fluorescence intensity in CX_3_CR1^GFP/GFP^ microglia, compared to CX_3_CR1^+/GFP^ microglia (Supplementary Figure 9A–D). There was no significant difference in the number of microglia or pericytes in the somatosensory cortex of 12‐week‐old NG2DsRed × CX_3_CR1^+/GFP^ mice compared to NG2DsRed × CX_3_CR1^GFP/GFP^ mice (Supplementary Figure 9E,F). Both CAM and PEM were present in both heterozygous and homozygous KO mice (Figure 6a,b), and upon quantification there was no significant difference in the proportion of microglia that were CAM or PEM in heterozygous versus homozygous KOs (CAM: heterozygous 42.4 ± 3.3% versus homozygous 43.6 ± 2.6%, *p* = .5435, Figure 6c; PEM: heterozygous 6.9 ± 1.1% versus homozygous 7.4 ± 0.8%, *p* = .4532, Figure 6d). To determine if the KO of CX_3_CR1 from microglia influenced vessel width, we also assessed the width of capillaries at pericytes with PEM and at vessels with CAM, finding no significant difference between heterozygous and homozygous KOs (CAM: heterozygous 4.7 ± 0.2 μm versus homozygous 4.9 ± 0.2 μm, *p* = .0626, Figure 6e; PEM: heterozygous 5.2 ± 0.5 versus homozygous 5.2 ± 0.4 μm, *p* = .9976, Figure 6f).

### 3.6 CAM and PEM are reduced in the Alzheimer's disease superior frontal gyrus

To assess whether there is a change in the prevalence of microglia and pericyte associations in AD, we assessed CAM, PEM, microglia and pericytes in post‐mortem tissue from the SFG of a human AD cohort. We first assessed the levels of amyloid and tau pathology in control and AD cases (Supplementary Figure 10A,B,D–E), finding significantly higher levels of MOAB‐2‐positive amyloid beta (Aβ) pathology (control 0.8 ± 0.6 vs. AD 2.9 ± 2.5 per mm^2^, *p* = .0083, Supplementary Figure 10G) and AT8‐positive hyperphosphorylated tau (control 0.0 ± 0.0 vs. AD 16.0 ± 7.6 per mm^2^, *p* < .0001, Supplementary Figure 10H) in AD cases compared to controls, supporting the plaque density and Braak scores in the pathology summary (Supplementary Table 1).

**FIGURE 7:**
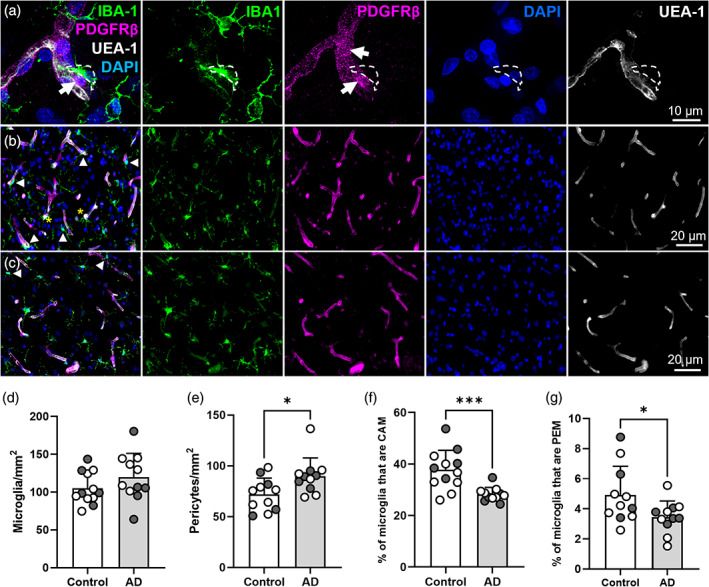
There are more pericytes in the superior frontal gyrus in Alzheimer's disease, but the proportion of microglia that are PEM and CAM is reduced. (a) Representative image of PDGFRβ‐positive pericytes (magenta), IBA1‐positive microglia (green), UEA‐1‐labeled vessels (white) and DAPI‐labeled nuclei (blue) from SFG of a human control brain (93 y.o. female). IBA1, PDGFRβ, DAPI and UEA‐1 channels are shown alone to the right of main image. White arrows identify pericyte soma. White dashed lines outline the position of a PEM. (b, c) Representative images of IBA1, PDGFRβ, DAPI and UEA‐1 combined, and split into individual channels, in the SFG of (b) a control brain and (c) an AD brain. White arrowheads denote CAM and yellow asterisks denote PEM. (d–g) Quantification of (d) microglia, (e) pericytes, (f) CAM and (g) PEM in the SFG of control (*n* = 11–12) and AD (*n* = 11) post‐mortem brains. (d, f, g) Analyzed with an unpaired parametric *t*‐test. (e) Analyzed with an unpaired nonparametric Mann–Whitney test. For all graphs, gray circles represent males and white circles represent females. Data are presented as mean ± SD. *p < .05; **p < .01.

Both microglia and pericytes could be visualized in the human brain and both CAM and PEM were identifiable in control and AD tissue (Supplementary Figure 1, Figures 1l and 7a–c, Supplementary Movies S5 and S11). There was no significant difference in the number of microglia in controls versus AD (control 105.2 ± 21.2 versus AD 119.8 ± 31.4 per mm^2^, Figure 7d). However, we observed a significant increase in the number of pericytes in the SFG of AD cases, compared to controls (control 71.9 ± 16.0 vs. AD 89.9 ± 17.9 per mm^2^, *p* = .0336, Figure 7e). Quantification of the proportion of microglia that were CAM and PEM showed they were present in similar proportions to mouse brain tissue sections (~30%–40% of microglia were CAM and ~4%–5% of microglia were PEM, Figures 7f,g). Despite the increased density of pericytes throughout the SFG in the AD brain, we observed a significant decrease in the proportion of microglia that were CAM and PEM in AD compared to controls (CAM: control 37.7 ± 7.6% vs. AD 28.2 ± 2.7%, *p* = .0008, Figure 7f; PEM: control 4.9 ± 1.9% vs. AD 3.4 ± 1.1%, *p* = .039, Figure 7g). Finally, we assessed microvessel density, observing no difference in the overall length of microvessels in AD brain sections compared to control brain sections (control 7559 ± 2197 μm/mm^2^ vs. AD 8528 ± 2199 μm/mm^2^, *p* = .303, Supplementary Figure 10C,F,I). Collectively, these data suggest that the SFG in AD exhibits an increase in pericyte density and a reduction in the association of microglia with capillaries and pericytes.

## 4 DISCUSSION

We identify and describe a spatially distinct population of microglia that dynamically associate with capillary pericytes in the mouse brain and spinal cord, and the human brain. For simplicity, we have named these cells pericyte‐associated microglia (PEM). Collectively, our data, combined with reports of similar interactions in the retina (Mills et al., 2021), suggest PEM are a ubiquitous feature of the CNS present at all levels of the capillary tree. Furthermore, we corroborate a previous report that found no difference in the prevalence of CAM between male and female mice (Bisht et al., 2021), and we also report no sex differences in PEM prevalence in adult mice.

It is important to note that we do not necessarily view PEM as a specific subtype of microglia. For the purposes of this study, the term PEM was purely a spatial definition. Future research may define specific features of PEM that will justify labeling them a subtype, but we cannot conclude this based on the findings in this study alone. As has been recently discussed, microglia have many layers of complexity (Paolicelli et al., 2022), and there is much we do not yet know about PEM. However, we do know that PEM spatially overlap with other vessel‐associated microglia, as the majority of PEM we analyzed would also be classified as CAM based on strict spatial definitions alone (Bisht et al., 2021). It is possible that PEM and CAM exist on the same continuum, especially considering CAM can migrate and may therefore move to and from pericyte cell bodies. We do note however that CAM may not be preferentially adjacent to pericyte cell bodies, as the proportion of microglia that are CAM outnumber the proportion of microglia that are PEM by at least 3‐fold, but by as many as 5‐fold in some regions of the brain. Furthermore, although most PEM are also CAM, there may be some distinguishing features of PEM compared with CAM that are not near pericytes. For example, in the adult brain over 80% of juxtavascular microglia remain adjacent to the vasculature over a 6 week period when imaged through cranial windows (Mondo et al., 2020). This finding was supported by Bisht et al. (2021) who identified ~70% of CAM were stable over a 4‐week imaging period (Bisht et al., 2021). These findings differ from our data where we identified that less than half (44%) of the PEM we tracked over 28 days remained adjacent to pericytes throughout the whole imaging period. These findings suggest PEM may be a uniquely motile subset of CAM.

Astrocyte endfeet almost completely cover endothelial cells, with only a few sites through which the processes of other cells can penetrate this coverage (Mathiisen et al., 2010). There is a growing body of data from electron microscopy studies showing that microglia are one cell type that can do this, directly contacting the basement membrane of blood vessels and thereby contributing to the glial limitans (Bisht et al., 2016, 2021; Császár et al., 2022; Haruwaka et al., 2019; Joost et al., 2019; Lassmann et al., 1991; Mathiisen et al., 2010; Mondo et al., 2020). In this position, microglia have been shown to form purinergic contacts with endothelial cells (Császár et al., 2022), and can form tight junctions with endothelial cells during inflammation (Haruwaka et al., 2019). The association of microglia cell bodies and processes with the basement membrane of capillaries also places them in position to interact with capillary pericytes. Importantly, one electron microscopy study found that the coverage of pericytes by astrocyte endfeet is incomplete, raising the possibility the basement membrane covering pericytes could be directly contacted by cells of the CNS without the interference of astrocyte endfeet (Mathiisen et al., 2010). Here, using both confocal and electron microscopy, we found evidence of direct microglia contact with the basement membrane covering pericyte cell bodies, without the interference of astrocyte endfeet. We did not however find evidence that pericytes with a PEM are unique in having less astrocyte coverage than those without a PEM. Instead, quantification of our AQP4 labeling suggested there is heterogeneity in AQP4‐positive astrocyte endfeet coverage of pericytes, irrespective of whether a pericyte has a microglia cell body directly opposed to it. This is not surprising, as astrocyte endfeet coverage of pericytes may be incomplete in areas where other cells of the CNS directly contact them.

Despite observing contact between microglia and the basement membrane covering pericytes, we do not yet know if they form tight junctions like those observed between microglia and endothelial cells during inflammation (Haruwaka et al., 2019). We also have not determined if there are specialized structures between them, akin to the peg‐and‐socket type structures pericytes form with endothelial cells (Ornelas et al., 2021), nor do we know how long contact points may last. In this study, we focused on cell body‐to‐cell body interactions, but microglia processes may also contact the basement membrane covering pericyte cell bodies and processes, which could provide additional sites for communication. This has recently been observed and quantified by others, with 83% of pericyte cell bodies contacted by microglia processes (Császár et al., 2022). Future studies will be needed to better understand the precise ultrastructural relationship between microglia and pericytes.

A number of signaling pathways have been described to connect pericytes and parenchymal cells, particularly astrocytes and neurons (Sweeney et al., 2016). One candidate pathway that may play a role in the association and function of microglia at blood vessels in the CNS is the fractalkine (CX_3_CL1)/CX_3_CR1 signaling pathway. A deficiency in the fractalkine receptor CX_3_CR1 delays the association of microglia with blood vessels in the developing brain (Mondo et al., 2020). Furthermore, in the brain and in retinal explants, the addition of CX_3_CL1 causes capillary constriction at regions of microglia contact (Mills et al., 2021). The precise cellular source of CX_3_CL1 that could reproduce these effects in vivo is not yet clear, but capillary pericytes do have the capacity to release it (Smyth et al., 2018). In this study, we found no change in the number of pericyte‐microglial associations in CX_3_CR1 KO mice, suggesting the CX_3_CL1/CX_3_CR1 axis may not be a major pathway for PEM recruitment or maintenance in the healthy adult mouse brain. Furthermore, we found no evidence that vessel width significantly changed beneath pericytes with a PEM with CX_3_CR1 KO. We found a trend toward increased vessel width at vessel segments with CAM in CX_3_CR1 KO mice, which would support the findings that the CX_3_CL1/CX_3_CR1 axis may be involved in regulating vessel tone, but this did not reach statistical significance. There is however a caveat with this dataset as vessel width was measured using a basement membrane marker, rather than a luminal marker, so we cannot rule out that vessel lumen diameter may have changed. These findings also do not rule out a role for the CX_3_CL1/CX_3_CR1 axis being involved in microglia‐pericyte signaling in disease conditions, especially considering pericytes increase the release of CX_3_CL1 in response to stimuli (Smyth et al., 2018). Other pathways such as the PANX1‐PR2Y12 signaling axis (Bisht et al., 2021; Császár et al., 2022) and the CCL5‐CCR5 signaling pathway (Haruwaka et al., 2019) have been implicated in the vascular functions of microglia (recently reviewed by Barkaway et al., 2022). These pathways may also play a role in recruitment and maintenance of microglia at pericyte locations in the healthy and injured brain.

There are many possible functions for the association between microglia and pericytes. A recent report suggests pericytes promote microglial proliferation and indirectly influence the role of microglia in neural stem cell differentiation (Hattori et al., 2022). Another logical hypothesis is that PEM are involved in regulating capillary tone indirectly via pericytes. In support of this hypothesis, recent studies have suggested microglia can influence CBF in health and disease (Bisht et al., 2021; Császár et al., 2022; Mills et al., 2021), but the mechanisms governing how they may do this are not yet understood. Pericytes are well established to control cerebral blood flow (Hall et al., 2014; Mishra et al., 2016; Neuhaus et al., 2017; Peppiatt et al., 2006; Sieczkiewicz & Herman, 2003; Yemisci et al., 2009), and signals received from other cells such as astrocytes have been shown to modulate capillary tone via pericytes (Mishra et al., 2016). We observed an increase in vessel width underneath both pericytes and CAM in the basal state and this increase was also observed when pericytes had a PEM. Furthermore, when a pericyte gained a PEM, blood vessel width increased beneath these pericytes. Conversely, when a pericyte lost a PEM, blood vessel width decreased. Combined, these results suggest that pericytes and their PEM may modulate blood flow. However, further studies are needed, particularly assessing evoked capillary responses at sites of PEM and CAM, or in disease states to confirm a specific effect of this interaction on blood flow modulation.

Microglia migrate to vessels in response to various noxious stimuli including stroke (Jolivel et al., 2015), traumatic brain injury (Grossmann et al., 2002), systemic inflammation (Haruwaka et al., 2019), laser‐induced vascular injury (Lou et al., 2016), and experimental autoimmune encephalomyelitis (Davalos et al., 2012; Joost et al., 2019). In one study microglia were found to cluster around pericytes in a model of epilepsy (Klement et al., 2018). Here, we observed the opposite phenomenon in the SFG of the AD brain, with a reduction in the proportion of microglia that were both CAM and PEM. Curiously, our data supports an earlier study, which found reduced microglia coverage within the neurovascular unit in the hippocampus and medial frontal gyrus of AD patients (Kirabali et al., 2020). It is possible microglia are being recruited to scavenge excess Aβ that is being deposited in the extracellular space, or that they are responding to tau pathology. Microglia have long been known to cluster around amyloid plaques and phagocytose Aβ (D'Andrea et al., 2004; Paresce et al., 1996), and microglia activation has been associated with tau pathology (Wang et al., 2022). A result of microglia responses to amyloid or tau pathology could be the loss of their physiological function at blood vessels and pericytes, which may contribute to functional decline in AD.

Our finding that pericyte number was greater in the SFG in AD compared to controls is contrary to other studies where a decline in the number of pericytes was observed in the frontal cortex and hippocampus in human AD (Sengillo et al., 2013), with an *APOE4* genotype appearing to exacerbate this pericyte loss (Halliday et al., 2016). Other studies have similarly found decreased pericyte coverage in the hippocampus and middle frontal gyrus as Braak stage increases (Kirabali et al., 2019, 2020). However, two studies have recently challenged the notion that pericyte death is a general feature of AD pathology. One study found pericyte density was 28% greater in the middle frontal gyrus of AD cases, compared to age‐matched controls (Fernandez‐Klett et al., 2020). A second study found a significant increase in the density of pericytes per length of vessels in the medial/dorso‐lateral frontal cortex of AD cases compared to controls (Ding et al., 2021). Our results corroborate these studies by showing higher pericyte numbers in AD compared to controls in an adjacent region, the SFG, and therefore provide further evidence that pericyte death is not necessarily a feature within every brain region in AD.

Our study has some limitations. For our human tissue analysis, we had limited AD tissue available to us and the tissue available was derived from the SFG, a less often studied region in AD literature. However, the SFG does exhibit AD pathology and we confirmed in our tissue that there was significantly more amyloid and tau pathology in the AD cases compared to the controls. So, the use of SFG tissue in this study allowed us to contribute new knowledge on the pathology of pericytes, microglia, CAM and PEM in a rarely studied brain region affected by AD. A further limitation of our human tissue analysis was that we have not quantified the spatial relationship between pericytes, microglia, CAM and PEM to AD pathology, to determine if AD pathology may be influencing CAM and PEM. This will be an important future direction.

Our studies with the NG2DsRed × CX_3_CR1^+/GFP^ model also have several limitations. Our use of cranial windows implanted over a craniotomy, as opposed to imaging through a thin‐skulled preparation, introduces the limitation that residual inflammation may have influenced the relationship between pericytes and microglia. Indeed, we observed a higher proportion of PEM in our cranial window data compared to our fixed tissue analysis, supporting this theory. We cannot however rule out that there were simply more PEM in this discrete region of the cortex because we manually selected ROIs that were enriched for PEM, independent of any possible inflammation induced by the cranial window surgery. We also have not uncovered a mechanism that controls the relationship between pericytes and microglia. Furthermore, we only conducted our analyses in young adult mice, so it would be interesting in future work to explore the changing relationships between microglia and pericytes in development and aging. Finally, our analysis primarily focussed on cell body associations. Previous works have indicated extensive coverage of pericytes by microglial processes and contact between microglial cell bodies and pericyte processes (Császár et al., 2022; Hattori et al., 2022; Mills et al., 2021), so understanding the relative importance of microglial cell body or cell process contacts to the functional relationship between pericytes and microglia will be an important future direction.

In summary, we have found that microglia can reside directly adjacent to pericytes in the healthy brain, and we have termed these PEM. PEM are consistent in their distribution throughout the brain and spinal cord regions assessed in this study in the healthy young adult mouse brain. They could serve several important functional roles including the modulation of blood flow and maintenance of the blood–brain‐barrier. However, in clinical AD the proportion of microglia that are PEM is reduced in the SFG, which may have implications for the physiological function of microglia at the vasculature. Our work provides a platform to begin understanding the functions and signaling mechanisms controlling the communication between pericytes and microglia, and how a breakdown in the associations may contribute to the development of AD and other neurological diseases.

## AUTHOR CONTRIBUTIONS

**Gary P. Morris**: conceptualization; data curation; validation; formal analysis; investigation; methodology; visualization; software; project administration; writing – original draft; review and editing. **Catherine G. Foster**: conceptualization; data curation; validation; formal analysis; investigation; methodology; visualization; software; writing – original draft; review and editing. **Jo‐Maree Courtney**: data curation; methodology; resources; software; visualization; writing – review & editing. **Jessica M. Collins**: investigation; resources; methodology; writing – review & editing. **Jake M. Cashion**: data curation; formal analysis; methodology; investigation; visualization; writing – review & editing. **Lachlan S. Brown**: methodology; software; validation; writing – review & editing. **David W. Howells**: conceptualisation; supervision; writing – review & editing. **Gabriele C. DeLuca**: conceptualisation; supervision; funding acquisition; writing – review & editing. **Alison J. Canty**: conceptualisation; supervision; methodology; writing – review & editing. **Anna E. King**: funding acquisition; resources; writing – review & editing; visualization; supervision. **Jenna M. Ziebell**: conceptualization; methodology; investigation; resources; writing – original draft; review and editing; supervision. **Brad A. Sutherland**: conceptualization; methodology; formal analysis; resources; writing – original draft; review and editing; visualization; supervision; project administration; funding acquisition.

## FUNDING INFORMATION

This research was supported by two NHMRC Boosting Dementia Fellowships (APP1137776, BAS and APP1136913, AEK) and an NHMRC project grant (APP1163384, BAS and GCD). The funding sources had no role in the study design, in the collection, analysis and interpretation of the data, in writing the report, or in the decision to submit the article for publication.

## CONFLICT OF INTEREST STATEMENT

No conflicts of interest to disclose.

## Supporting information


**Data S1** Supporting information

Video S1

Video S2

Video S3

Video S4

Video S5

Video S6

Video S7

Video S8

Video S9

Video S10

Video S11

## Data Availability

The data that support the findings of this study are available from the corresponding author upon reasonable request.
