# Supplementary material for: Microglia directly associate with pericytes in the central nervous system
Source: Glia. 2023 Mar 30;71(8):1847–69. doi: 10.1002/glia.24371 (PMC10952742; doi:10.1002/glia.24371)
Supplement: Supplementary file 1 — Data S1 Supporting information [file GLIA-71-1847-s009.docx]

**SUPPLEMENTARY MATERIAL**

**Microglia directly associate with pericytes in the central nervous system**

Gary P. Morris^1†^, Catherine G. Foster^1†^, Jo-Maree Courtney^1^, Jessica M. Collins^2^, Jake M. Cashion^1^, Lachlan S. Brown^1^, David W. Howells^1^, Gabriele C. DeLuca^3^, Alison J. Canty^2,4^, Anna E. King^2^, Jenna M. Ziebell^2^ and Brad A. Sutherland^1^

**^†^These authors contributed equally to this work.**

**Author affiliations:**

1. Tasmanian School of Medicine, College of Health and Medicine, University of Tasmania, Hobart, Tasmania, Australia.

2. Wicking Dementia Research and Education Centre, College of Health and Medicine, University of Tasmania, Hobart, Tasmania, Australia.

3. Nuffield Department of Clinical Neurosciences, University of Oxford, Oxford, UK.

4. Global Brain Health Institute, Trinity College, Dublin, Ireland.

**Correspondence to:** A/Prof Brad Sutherland

Full address: Medical Sciences Precinct, 17 Liverpool Street, Hobart, TAS 7000, Australia, Ph: +61 3 6226 7634; Email: [brad.sutherland@utas.edu.au](mailto:brad.sutherland@utas.edu.au)

Or: Dr. Gary Morris

Full address: Medical Sciences Precinct, 17 Liverpool Street, Hobart, TAS 7000, Australia, Ph: +61 3 6226 4851; Email: [gary.morris@utas.edu.au](mailto:gary.morris@utas.edu.au)

**Supplementary Methods**

**Quantifying the proportion of oligodendrocyte progenitor cells and pericytes that are positive for NG2DsRed**

To quantify the proportion of PDGFRα and PDGFRβ positive cells (markers of oligodendrocyte progenitor cells (OPCs) and pericytes, respectively) that were co-positive for NG2DsRed, three male hemizygote NG2DsRed transgenic mice (11 to 20-weeks-old, The Jackson Laboratory stock #008241) were given a lethal intraperitoneal injection of pentobarbitone (150 mg/kg) and immediately transcardially perfused with heparinised PBS followed by 4% paraformaldehyde (PFA; pH 7.4). Whole brains were cut coronally at lambda, with the portion anterior from lambda prepared for cryosectioning as previously described (Courtney et al., 2021). Forty μm sections were cut coronally (at −18°C) using a cryostat and stored free floating in 1× PBS with 0.01% sodium azide at 4°C.

Two tissue sections from each brain (~Bregma −1.10 mm (Allen Reference Atlas)) were permeabilised in PBS with 0.3% Triton X-100 for 40 min at room temperature (RT), then incubated with serum free protein block (Dako, Cat# X090930-2) for 1h at RT. One set of sections was incubated overnight with goat-anti-PDGFRα (1:500, R&D Systems, Cat# AF1062, RRID:AB_2236897), with gentle agitation. A second set of sections was incubated overnight at 4°C with rat-anti-PDGFRβ (1:200, ThermoFisher, Cat# 14-1402-82, RRID: AB_467493), with gentle agitation. All sections were then rinsed with PBS (3x 5 min). PDGFRα and PDGFRβ labelled sections were then incubated in donkey-anti-goat 647 (1:1000; ThermoFisher, Cat# A21447, RRID: AB_2535864), or goat-anti-rat 647 (1:1000; ThermoFisher, Cat# A21247, RRID: AB_141778), respectively, in antibody diluent for 2h at RT. A *Lycopersicon esculentum* (tomato) lectin stain (LEL) was included in this secondary step alongside both antibodies (1:500, Sigma-Aldrich, Cat#L0401). All sections were washed once in PBS with DAPI (1:10,000, Invitrogen, Cat#D3571, RRID: AB_2307445, 5 min), then rinsed in PBS (2 x 5 min). Sections were then treated with Trueblack according to the manufacturers instructions, with modification (treatment was for 5 min, not 1 min). Sections were then rinsed briefly in PBS, then washed in PBS (2 x 2 min), mounted onto slides (Dako, catalog #K802021-2), briefly airdried and cover slipped with Prolong Gold with DAPI (Life Technologies, Cat# P36935). Sections were imaged at 20x magnification across five focal planes, spaced 3 μm apart, using the extended focal imaging setting. Optimum exposure times were determined manually and kept consistent for all images in each cohort.

Using QuPath 3.2, 800µm x 800µm regions of interest (ROIs) were drawn in the somatosensory cortex (one ROI per hemisphere). DAPI-positive nuclei were automatically detected within these boxes. To determine the proportion of NG2DsRed positive cells that were OPCs, or pericytes, all NG2DsRed positive nuclei were counted and then manually assessed as either PDGFRα or PDGFRβ positive. A separate analysis was conducted to determine the proportion of PDGFRα or PDGFRβ positive cells that were NG2DsRed positive. To do this, every DAPI positive nucleus was manually assessed and deemed either PDGFRα positive, PDGFRβ positive, or negative for both markers. This assessment was conducted with the NG2DsRed channel turned off to reduce bias. PDGFRα or PDGFRβ positive cells were then manually classified as either NG2DsRed positive or NG2DsRed negative.

Perivascular fibroblasts on large vessels can be positive for PDGFRα, NG2DsRed and PDGFRβ (Bonney et al., 2022). Furthermore, vascular smooth muscle cells (VSMCs) are positive for both PDGFRβ and NG2DsRed (Attwell et al., 2016). So that these cells did not interfere with the quantification, the vessel stain (LEL) combined with the morphology of VSMCs was used to identify large vessels on which these cells exist, and they were excluded from the counts.

**Mathematically modelling how frequently microglia would be classified as PEM by chance**

To determine if the proportion of microglia that were pericyte-associated microglia (PEM) was more than what would be expected by random chance, we created a mathematical model using the following protocol. First, for each simulation, we chose the number of microglia/mm^2^ and pericytes/mm^2^ based on data we had obtained from ten biological replicates. For example, for one of the replicates there were 246 microglia/mm^2^ and 155 pericytes/mm^2^. In this example, all 246 microglia and 155 pericytes were given random X and Y coordinates between 0 and 1000 (using the RAND function in excel), to simulate each microglia and pericyte being placed at random within a 1000 x 1000 μm space. For each individual microglia, we then compared the XY coordinates to the XY coordinates of every pericyte (using Pythagoras’ theorem), to determine how many individual microglia were within 10 μm of a pericyte. We repeated this process 10,000 times to determine the average number of microglia that were <10 μm from a pericyte across 10,000 iterations. This allowed us to determine the percentage of microglia that would be expected to be a PEM by random chance. We then repeated the protocol using microglia and pericyte cell densities derived from the other nine biological replicates, to model the number of microglia that would be predicted to be PEM by random chance in each biological replicate. Finally, we repeated the process using the average number of microglia (244/mm^2^) and pericytes (153/mm^2^) from our ten biological replicates that were used to quantify the percentage of PEM in the somatosensory cortex (Fig. 1E). This enabled us to produce Supplementary Fig. 4, which compares the distribution of predicted PEMs across 10,000 replicates using the mean number of microglia and pericytes, to the distribution obtained from our ten biological replicates.

**Tissue collection, processing and quantification of PEM in different regions of the mouse brain and spinal cord**

**Quantifying CAM and PEM in different regions of the mouse brain**

For PEM quantification across multiple brain regions in NG2DsRed x CX_3_CR1^+/GFP^ tissue, mounting and drying procedures were the same as detailed in the methods section, however three sections per brain (Bregma 0.7 (to analyse caudate putamen), -0.1 (to analyse the somatosensory cortex) and -2.0 (to analyse hippocampus, thalamus and hypothalamus) were slide mounted. Sections were washed in PBS with 0.1% Tween20. Tissue was then cover slipped with Prolong Gold antifade reagent with DAPI (Life Technologies, Cat#P36935). These sections were imaged in a single focal plane at 40x magnification using a VS120 Virtual Slide System (Olympus). Optimum exposure times for DAPI, DsRed and GFP were determined manually and kept consistent for all images in each cohort.

QuPath 3.0 was used to quantify the prevalence of PEM in different regions. The caudate putamen, somatosensory cortex, hippocampus, hypothalamus and thalamus, were manually traced using the Allen Brain Atlas as a guide (Allen Reference Atlas). Areas of tissue with processing issues (e.g. out-of-focus areas, tissues rips and folds) and large NG2DsRed-positive smooth muscle cells were manually removed from regions of interest (ROIs). Automatic detection of DAPI-positive nuclei was optimised as previously described (Courtney et al., 2021, 2022), with modification: 250 x 250 μm boxes were used to optimise DAPI detection parameters; and combinations of 15 different thresholds, six different sigmas and three different background radii were tested using a custom-built script. The optimised nuclei detection parameters were applied using a custom script (Courtney et al., 2021, 2022), in combination with a manual threshold, that identified NG2DsRed and CX_3_CR1^+/GFP^-positive nuclei. Nuclei that were inaccurately assigned (i.e. false positives, or false-negatives) were manually re-assigned. Cells with overlapping nuclei that were both NG2DsRed and CX_3_CR1^+/GFP^-positive were classified as dual positive. After cell classification was complete, the ‘Detect Centroid Distances 2D’ function was used to calculate the nearest nuclei of a different classification (i.e. the nearest microglia to each pericyte, or the nearest pericyte to each microglia). Dual-classified cells were deemed to be pericytes or microglia <5 μm from a microglia or pericyte, respectively. The numbers of pericytes and microglia for each region, and the cell proximity data were exported as .tsv files and analysed in Microsoft Excel and GraphPad Prism 9.3.1.

**Quantifying CAM/PEM prevalence in the spinal cord**

Spinal cords from NG2DsRed x CX_3_CR1^+/GFP^ mice were postfixed for 1.5h in 4% PFA, transferred to 30% sucrose in PBS until they sank, embedded in cryomatrix embedding resin, flash frozen, then stored at -80°C. Transverse 40 µm thick sections were cut using a cryostat and placed free floating in PBS. Only a subpopulation of spinal cords from female mice *(n =* 3) were available for analysis.

For vessel labelling of spinal cords, sections from T1-T13, as per the Atlas of the Mouse Spinal Cord (Watson et al., 2009), were slide mounted, air dried, and incubated in isolectin GS-IB4 (IB4, 1:100) diluted in PBS with 0.1% Tween20 for 2h at RT. Sections were washed with PBS (5 min), incubated with PBS containing DAPI (1:10,000, Invitrogen, Cat#D3571, RRID: AB_2307445) for 10 min, washed in PBS (3 x 5 min) and coverslipped in fluorescent mounting media (Agilent, Cat#S3023).

Microglia, pericytes, capillary-associated microglia (CAM) and PEM were imaged and quantified manually in spinal cords from NG2DsRed x CX_3_CR1^+/GFP^ using the same protocols that were employed to image and manually count cells in the somatosensory cortex (see main methods section). Grey matter from the thoracic region of NG2DsRed x CX_3_CR1^+/GFP^ spinal cords was traced to create ROIs and quantification was made across the entirety of these ROIs. Microglia, pericytes, CAM and PEM were manually identified within the ROIs using the same protocol employed to manually quantify the somatosensory cortex (see main methods section).

**Quantification of GFP fluorescence intensity in** **NG2DsRed x CX_3_CR1^+/GFP^ vs. NG2DsRed x CX_3_CR1^GFP/GFP^ tissue**

QuPath 3.2 was used to quantify the fluorescence intensity of GFP-positive microglia in NG2DsRed x CX_3_CR1^+/GFP^ vs. NG2DsRed x CX_3_CR1^GFP/GFP^ tissue. First, 700 x 700μm boxes were placed in the somatosensory cortex. Using the positive cell detection function, DAPI-positive nuclei were automatically detected within these boxes. Nuclei positive for GFP were detected using the mean GFP intensity within DAPI-positive nuclei. Nuclei were deemed GFP-positive if they were above a manually selected threshold (800 arbitrary units). After cell detection was complete, the fluorescence intensity of every GFP-positive cell from each replicate was exported and analysed in Microsoft Excel. The mean intensity of all GFP-positive cells in each replicate was determined and used to compare GFP expression intensity in cells from NG2DsRed x CX_3_CR1^+/GFP^ vs. NG2DsRed x CX_3_CR1^GFP/GFP^ tissue.

**Assessment of amyloid beta pathology, tau pathology and blood vessel length in human brain sections**

Two separate sets of superior frontal gyrus (SFG) tissue sections were immunohistochemically labelled to quantify amyloid beta (Aβ) and tau pathology. Aβ and tau pathology were labelled using the same protocol outlined in the main text for labelling microglia and pericytes in human tissue sections (section 2.5.2), with modification. For Aβ labelling, tissue sections were not treated with citric acid antigen retrieval buffer. Instead, after first incubating in PBS with 0.3% Triton X-100 for 1h at RT, they were transferred to 98/100% formic acid (BDH Chemicals, Product No. 10115) for 8 min on a shaker at RT. Sections were then washed with PBS (6 x 5 min), before incubating in serum free blocking solution (Dako, Cat# X090930-2) for 1h at RT. The primary antibody for Aβ labelling was mouse anti-Aβ (MOAB-2, 1:1000; EMD Millipore, Cat# MABN254, RRID: AB_2895168) and sections were incubated with this antibody for two nights at 4°C. For tau labelling, tissue sections were treated the same as described for pericyte and microglia labelling in the main text (section 2.5.2), before adding mouse-anti-Phospho Tau (AT8, 1:500; Thermofisher, Cat# MN1020, RRID: AB_223647). Sections were incubated with the primary antibody for two nights at 4°C. For secondary labelling, both Aβ and tau labelled tissue were incubated in donkey-anti-mouse 488 (1:1000; Thermofisher, Cat# A-21202, RRID: AB_141607) and blood vessels were labelled with UEA-1 594 (1:1000; Vector Laboratories, Cat# DL-1067), which was added at the same time as the secondary antibody. Other than these modifications, all other steps of the protocol for both Aβ and tau labelling were the same as for microglia and pericyte labelling, as described in the main text.

Tau pathology was manually quantified using QuPath 4.2 according to the same protocol described for microglia and pericyte quantification in human tissue sections, in the main text, with modification. AT8 positive cells containing a DAPI positive nucleus were counted in ten full 500 x 500 μm squares. Aβ pathology in grey matter ROIs for each case was quantified in QuPath 4.2 using an automated pixel classifier to detect Aβ pathology labelled with MOAB-2. The classifier was set to moderate resolution (2.58μm/px) and a Gaussian prefilter was applied. MOAB-2 labelled Aβ pathology was deemed positive above a threshold of 1000, which enabled the determination of total Aβ positive area in each ROI.

Blood vessel length was quantified from UEA-1 labelled vessels using FIJI-ImageJ. Three 500 x 500 μm ROIs were exported for each tissue section from QuPath 3.0 to FIJI-ImageJ to build classifiers in the Trainable WEKA Segmentation plugin (Arganda-Carreras et al., 2017). Individual classifiers were built by annotating positive signal (i.e. UEA-1 594 representing blood vessels) and negative signal (i.e. background) on each of the three squares. Using individual classifiers for each replicate, the UEA-1 signal was then segmented in the same 20 boxes used for assessment of microglia and pericytes and a custom-built macro (modified from (Morris et al., 2023)) was applied to analyse vessel length using the plugins Skeletonize (2D/3D) and Analyze Skeleton (Arganda-Carreras et al., 2010). The researcher was blinded to case type throughout experimental procedures and analysis.

**Supplementary Results**


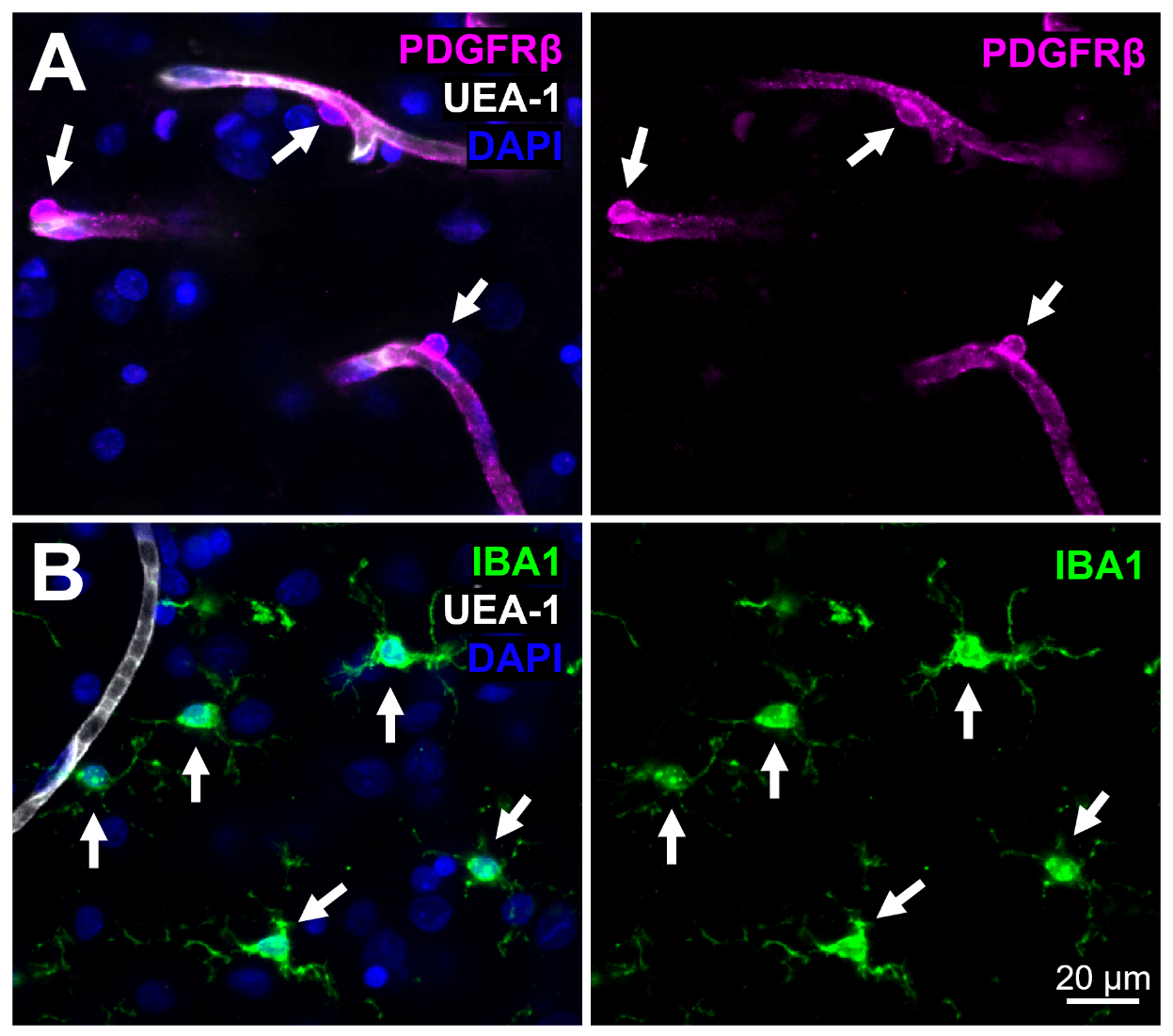


**Supplementary Figure 1. PDGFRβ and IBA1 labelling in human superior frontal gyrus.**

**(A)** Representative image of PDGFRβ-positive pericytes (magenta), UEA-1-labelled vessels (white) and DAPI-labelled nuclei (blue) from the superior frontal gyrus (SFG) of a human AD brain (86 y.o. male). Panel to the right shows the PDGFRβ channel alone. **(B)** Representative image of IBA1-positive microglia (green), UEA-1-labelled vessels (white) and DAPI-labelled nuclei (blue) from the SFG of a human AD brain (86 y.o. male). Panel to the right shows the IBA1 channel alone.


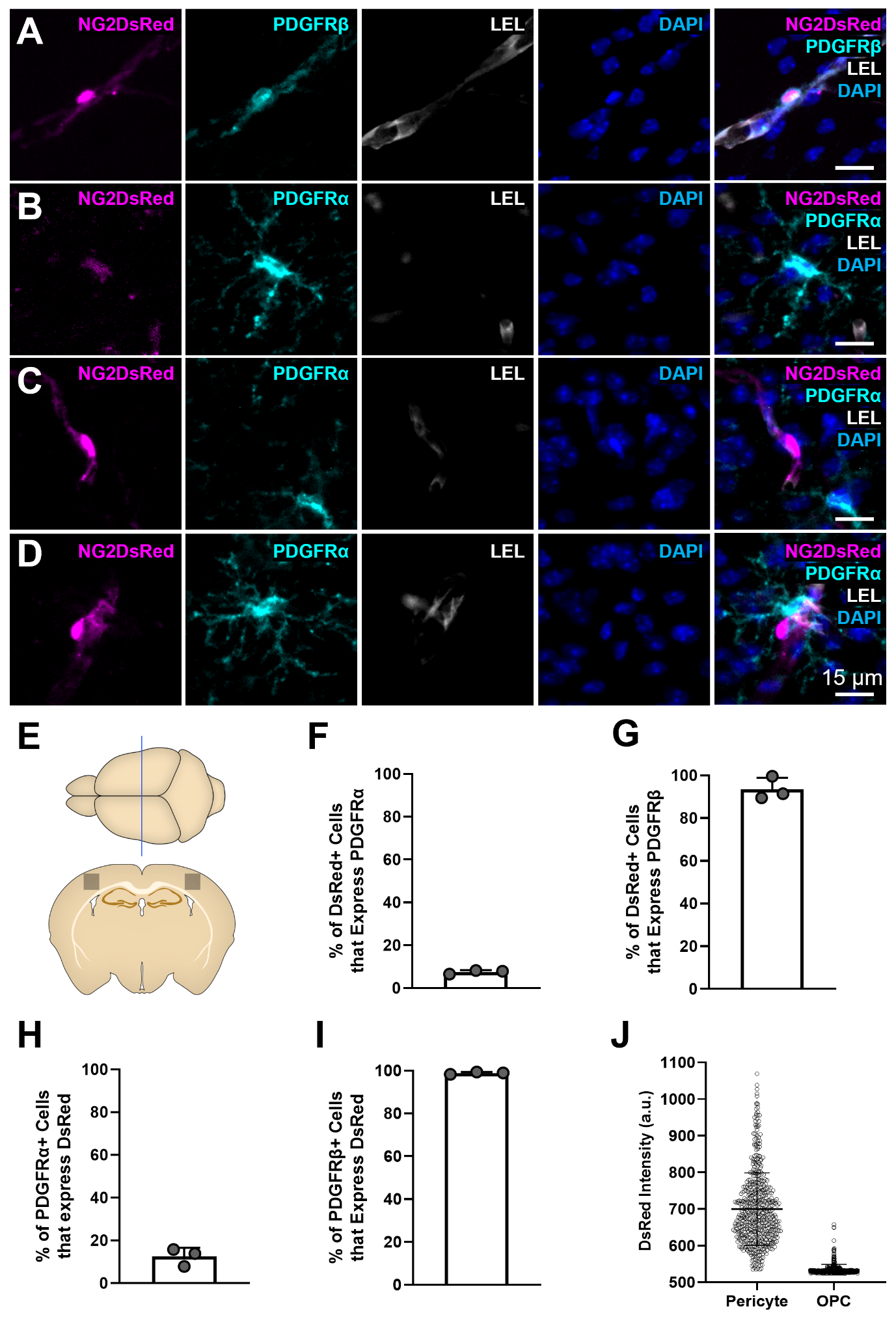


**Supplementary Figure 2. Few PDGFRα-positive oligodendrocyte progenitor cells express NG2DsRed**

**(A)** Representative image of an NG2DsRed-positive pericyte (magenta) expressing PDGFRβ (cyan). **(B)** Representative image of a weakly NG2DsRed-positive oligodendrocyte progenitor cell (OPC) expressing PDGFRα (cyan). **(C)** Representative image of an NG2DsRed-positive pericyte not expressing PDGFRα. **(D)** Representative image of an NG2DsRed-positive pericyte adjacent to a vessel/pericyte-associated PDGFRα-positive OPC. **(E)** Schematic of region analysed within the somatosensory cortex of NG2DsRed mice (Bregma -1.10 mm (Allen Reference Atlas)). **(F)** Percentage of NG2DsRed-positive cells co-positive for PDGFRα (*n* = 3 males per group). **(G)** Percentage of NG2DsRed-positive cells co-positive for PDGFRβ (*n* = 3 males per group). **(H)** Percentage of PDGFRα-positive cells co-positive for NG2DsRed (*n* = 3 males per group). **(I)** Percentage of PDGFRβ-positive cells co-positive for NG2DsRed (*n* = 3 males per group). **(J)** DsRed intensity of individual PDGFRα-positive OPCs and PDGFRβ-positive pericytes (*n* = 451 individual OPCs and 529 individual pericytes, *n* = 3 males per group). Tomato lectin (LEL) is in white and DAPI is in blue in all images. Data are presented as mean ± SD.


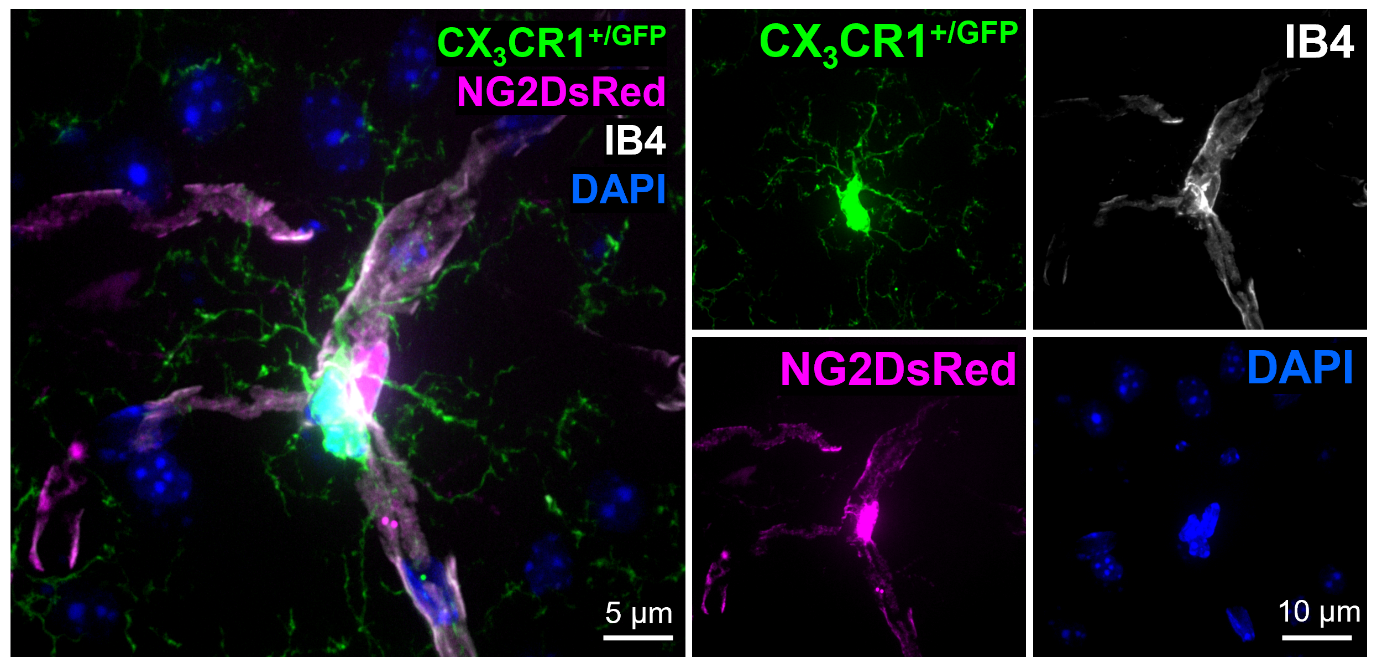


**Supplementary Figure 3. Pericyte-associated microglia curve around pericytes**

**Left image:** Representative example of a pericyte-associated microglia (PEM) morphologically curved around a pericyte in the somatosensory cortex. **Right images:** Image of the left panel split into channels of green CX_3_CR1^+/GFP^-positive microglia, white IB4-labelled vessels, magenta NG2DsRed-positive pericytes and blue DAPI-labelled nuclei.

**
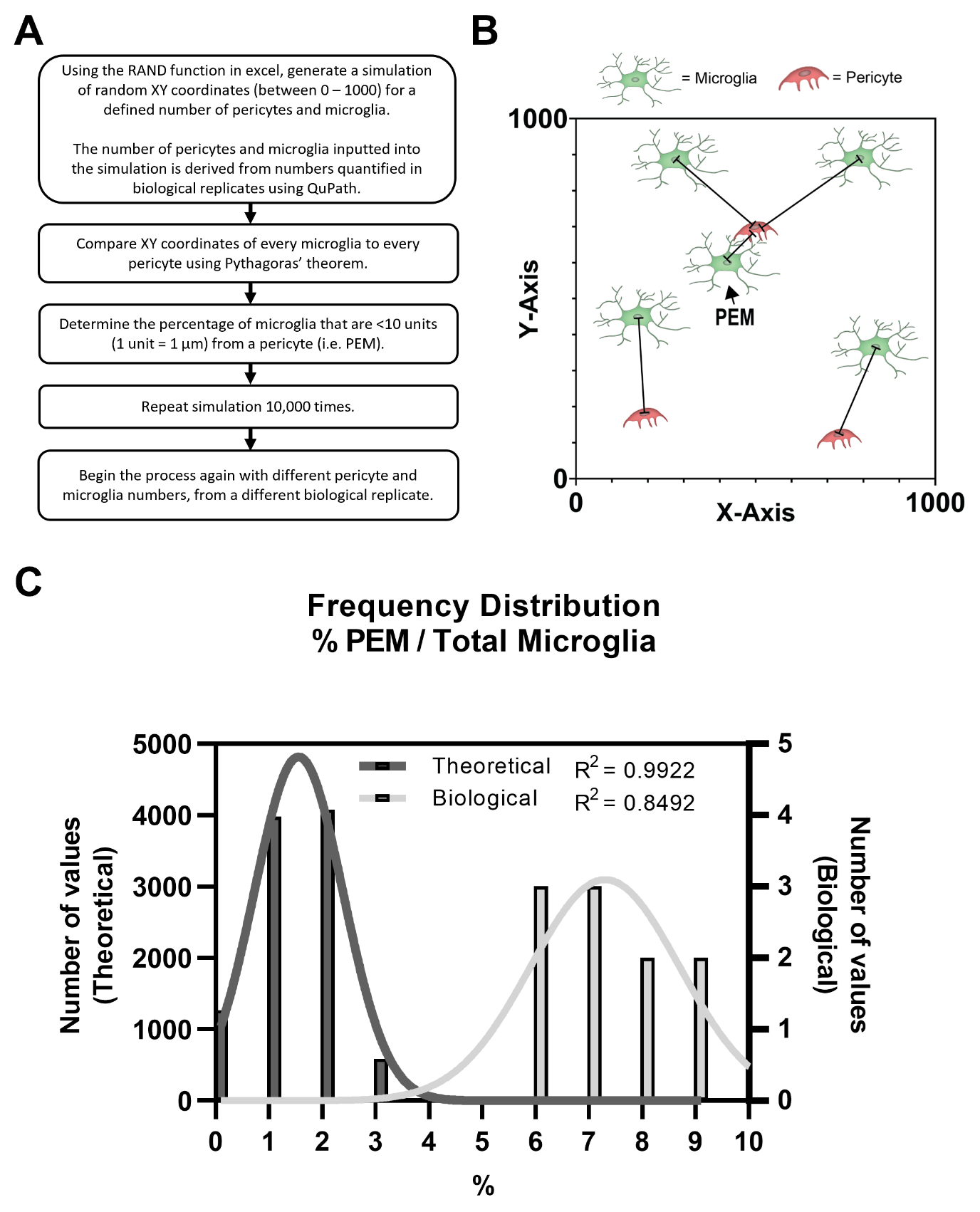
**

**Supplementary Figure 4. Mathematically modelling how frequently microglia would be classified as PEM by chance**

**(A)** Flow diagram describing the mathematical modelling method used to model the random chance of a microglia being within 10 µm of a pericyte in a 1000 x 1000 μm space. **(B)** Visual representation of the model described in (A). Black lines represent closest pericyte to each microglia. Black arrow identifies a PEM. **(C)** Histogram showing the frequency distribution (*n =* 10,000 iterations, left Y-axis, derived from a simulation that predicted PEM percentage using the average number of microglia and pericytes/mm^2^ derived from ten biological replicates. This is compared to a histogram showing the frequency distribution of the percentage of microglia that were found to be PEM through manual quantification of the same ten biological replicates values (right Y-axis). The x-axis is 1% bins. The lines of best fit show gaussian non-linear regressions and the R squared values represent the goodness-of-fit.


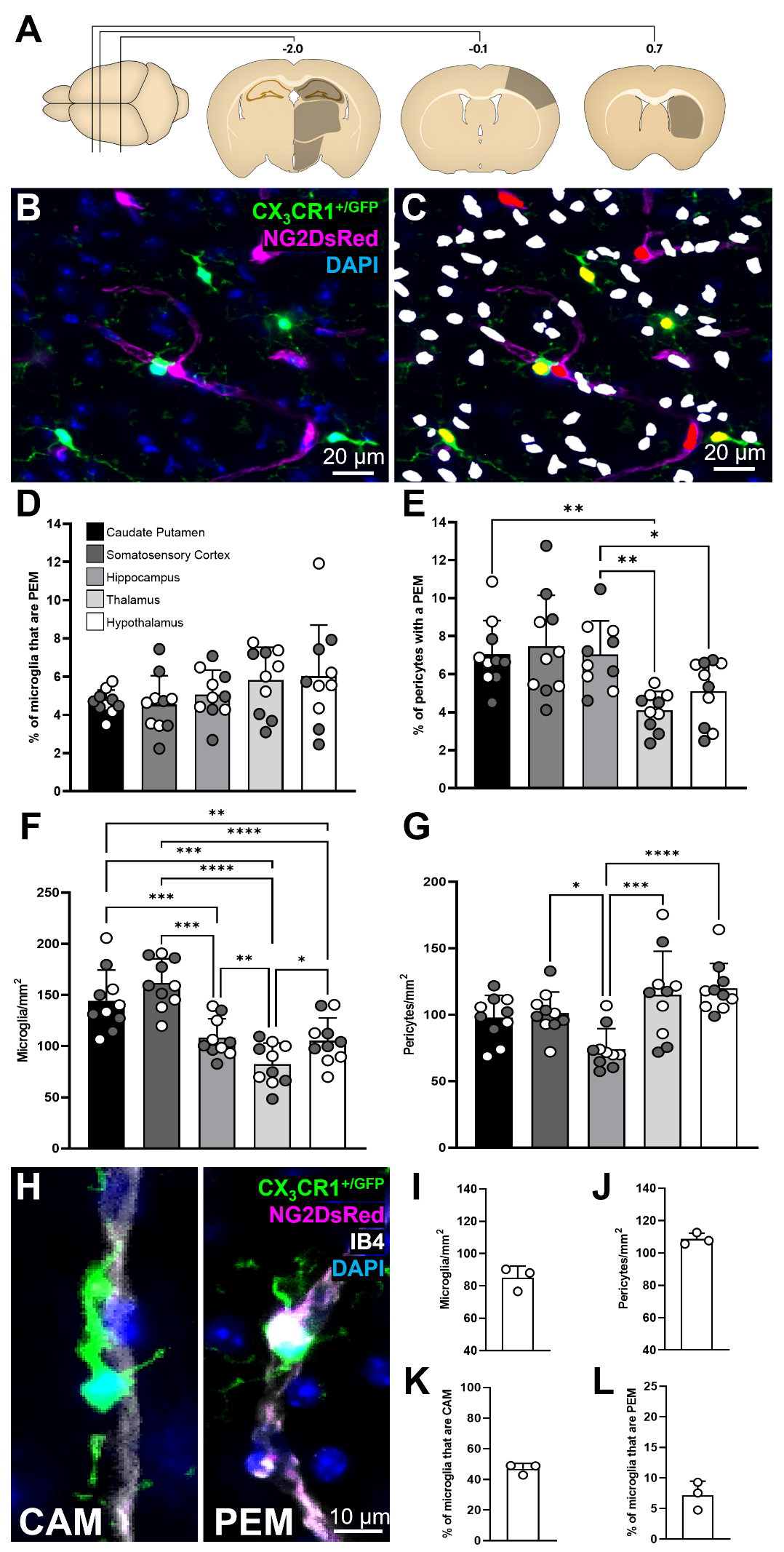


**Supplementary Figure 5. Pericyte-associated microglia are a ubiquitous feature of the central nervous system**

**(A)** Schematics of brain regions analysed for PEM prevalence in NG2DsRed x CX_3_CR1^+/GFP^ mice; the caudate putamen (Bregma 0.7), the somatosensory cortex (Bregma -0.1) and the hippocampus, thalamus and hypothalamus (Bregma -2.0) (Allen Reference Atlas) **(B-C)** Representative images of NG2DsRed x CX_3_CR1^+/GFP^ tissue (B) pre- and (C) post-cell classification using QuPath. DAPI-positive nuclei that were not classified as microglia or pericytes are labelled white. The nuclei of microglia are labelled yellow and the nuclei of pericytes are labelled red. **(D-E)** Quantification of (D) the proportion of microglia that are PEM and (E) the proportion of pericytes with a PEM in different regions of the brain in male (*n* = 5) and female (*n* = 5) mice. **(F-G)** Quantification of (F) microglia and (G) pericyte numbers in the caudate putamen, somatosensory cortex, hippocampus, thalamus and hypothalamus (*n* = 10, 5 male and 5 female). (D) Comparisons were made with a Friedman’s test (Friedman statistic = 9.04, *p*=0.0601). (E-G) Comparisons were made with a parametric repeated measures one-way ANOVA. (E) Brain region: F(2.5, 22.8) = 6.979, *p*=0.0026, (F) Brain region: F(2.5, 22.7) = 38.5, *p*<0.0001 (G) Brain region: F(1.8, 16.4) = 16.1, *p*=0.0002, all with Tukey’s *post hoc* test  **(H)** Representative image of a CAM (left) and a PEM (right) in the spinal cord of NG2DsRed x CX_3_CR1^+/GFP^ mice. **(I-J)** Quantification of (I) microglia and (J) pericyte numbers in the thoracic region of the spinal cord in female mice (*n* = 3). **(K-L)** Quantification of (K) the proportion of microglia that are CAM and (L) the proportion of microglia that are PEM in the spinal cord in female mice (*n* = 3). For all graphs, grey circles represent males and white circles represent females. Data are presented as mean ± SD. **p* < 0.05, ***p* < 0.01, *** *p* < 0.001, **** *p* < 0.0001.


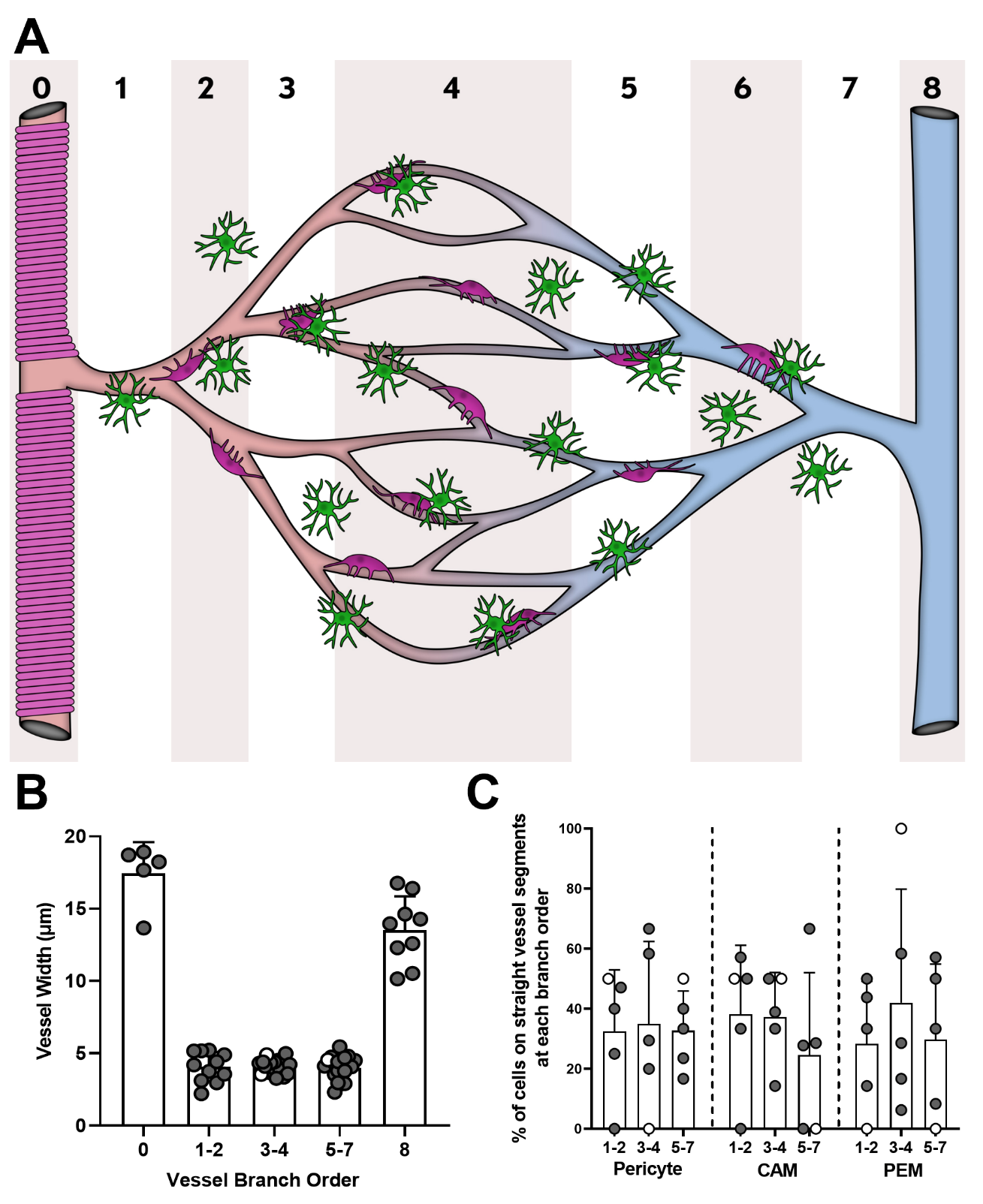


**Supplementary Figure 6. CAM and PEM are present at all branching orders of capillaries**

**(A)** Schematic showing the vascular tree with both penetrating arteriole and ascending venule, which are linked via the capillary bed. (0) Penetrating arteriole diving down into the parenchyma from the surface of the brain. (1) Penetrating arteriole (0th order) branching off to form a capillary (first order). (2-6) Higher order capillaries branching. (7) Seventh order capillary converging on the ascending venule. (8) Ascending venule rising from the parenchyma to the surface of the brain. Vascular smooth muscle cells are represented by the magenta rings on the penetrating arteriole. Pericytes are represented by the magenta cells on capillaries. Microglia are represented by the green cells with some interacting with vessels (CAM) and pericytes (PEM). **(B)** Vessel diameter measurements at each branching order of the vascular tree. **(C)** Percentage of total CAM, PEM and pericytes on different levels of the vascular tree when on straight vessel segments (*n* = 5, four male and one female). For all graphs, grey circles represent males and white circles represent females. Data are presented as mean ± SD.


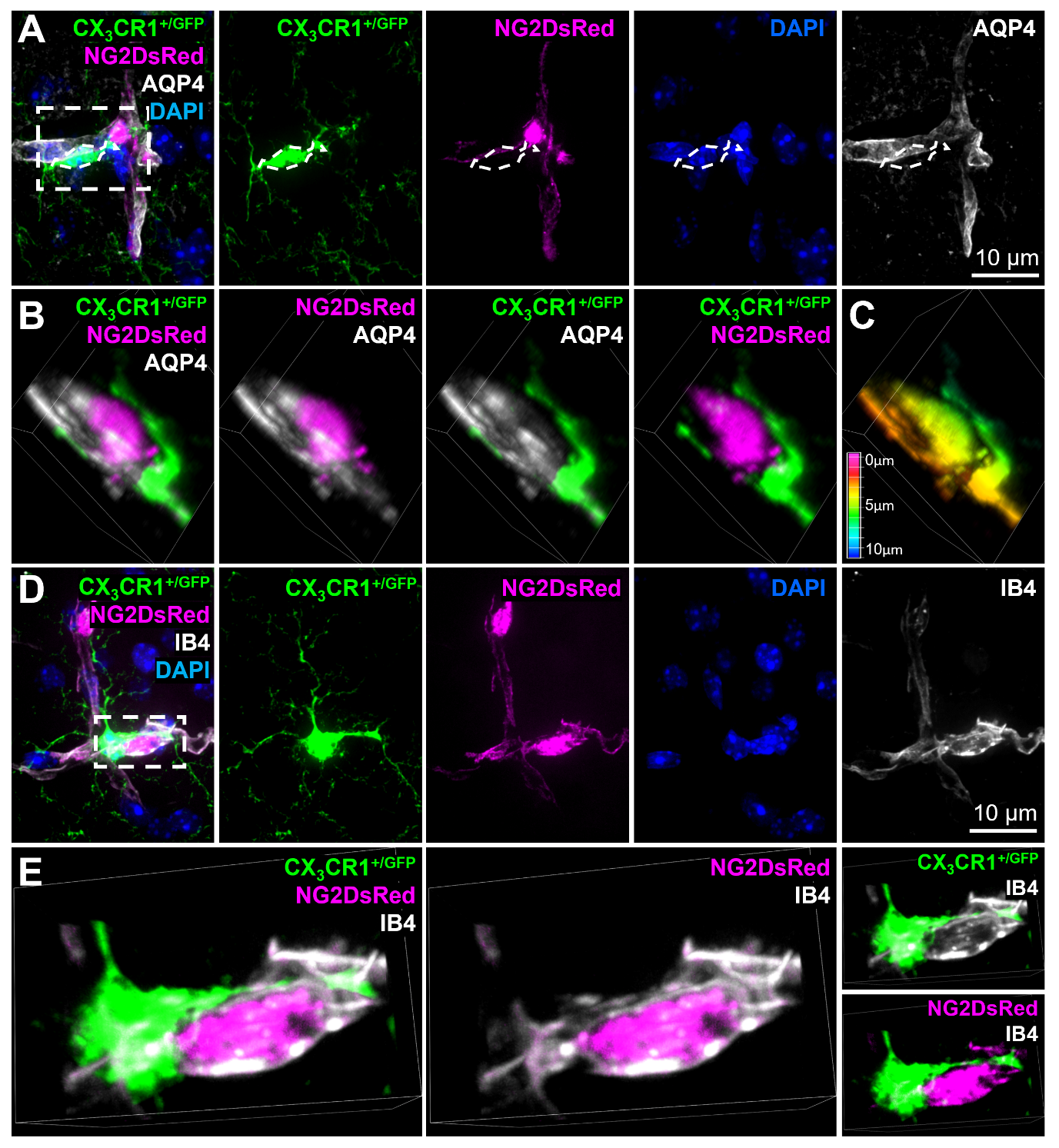


**Supplementary Figure 7. Basement membrane resides between pericyte-associated microglia and pericytes**

**(A)** Representative example of AQP4 coverage lacking at the site in which the process of a microglia is reaching around a pericyte in the somatosensory cortex (~Bregma -1.10). NG2DsRed-positive pericyte (magenta), CX3CR1+/GFP-positive microglia (green), AQP4-labelled astrocyte endfeet (white) and DAPI-labelled nuclei (blue) are shown. Images showing each fluorescent channel alone are to the right of the main image. **(B)** Magnification of dashed box in (A), rotated to illustrate the association between the pericyte soma and a microglial process at an area lacking AQP4 labelling. Each panel is rotated identically. **(C)** Depth shading of image in (A) illustrating the microglia process is in the same plane as the pericyte but AQP4 labelling is below the plane of the pericyte and microglia process. All images were derived from 12-week-old NG2DsRed x CX3CR1+/GFP mice using confocal microscopy. **(D)** Representative example of a pericyte-associated microglia extending a process around a pericyte (this image is also in Fig. 4A). This image highlights that the basement membrane sits between the microglial cell body and process and the pericyte soma. NG2DsRed-positive pericyte (magenta), CX_3_CR1^+/GFP^-positive microglia (green), IB4-labelled vessels (white) and DAPI-labelled nuclei (blue) are also shown in single channels. **(E)** Magnification of dashed box in (D), rotated to illustrate the presence of the basement membrane (IB4, white) between the microglia (green) and pericyte (magenta). This figure is also in Fig. 4B. Pericyte and basement membrane channels alone, microglia and basement membrane channels alone, and microglia and pericyte channels alone, are also shown. All images were derived from 12-week-old NG2DsRed x CX_3_CR1^+/GFP^ mice using confocal microscopy.

**
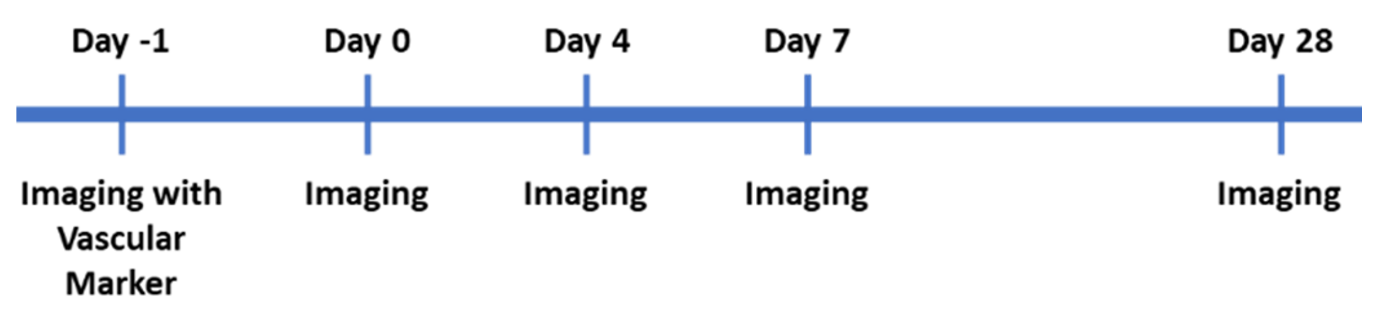
**

**Supplementary Figure 8. Schematic of two-photon imaging days in which PEM were defined and then tracked over a 28-day period.**

NG2DsRed x CX_3_CR1^+/GFP^ mice underwent cranial window implantation, and then recovery for 2 weeks. Initial imaging (Day -1) included tracing the vasculature with FITC-dextran injected intravenously to confirm pericyte and microglia locations adjacent to the vasculature. Subsequent imaging sessions to track PEM were conducted on days 0, 4, 7 and 28, with no vascular tracing.

**
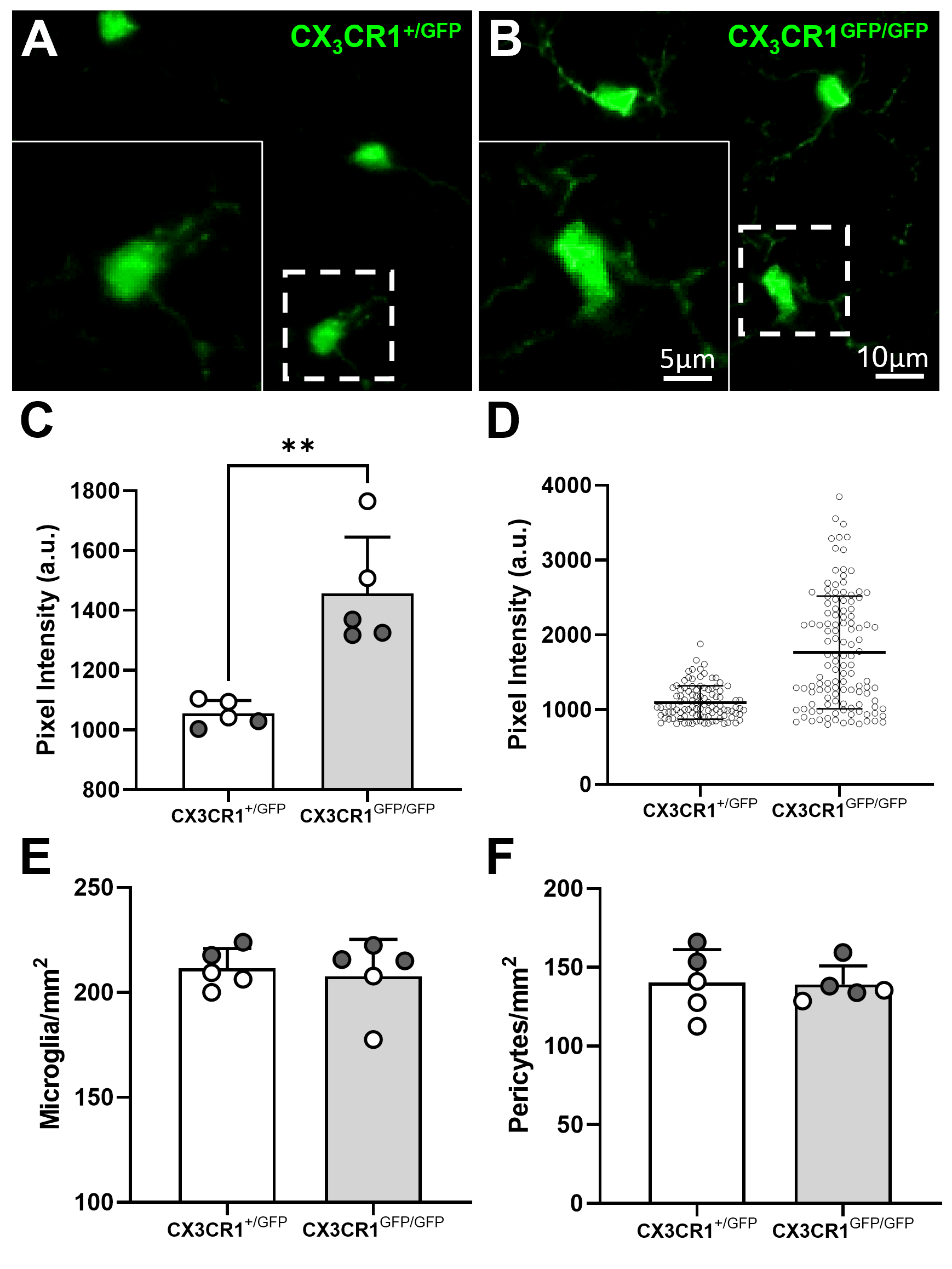
**

**Supplementary Figure 9. GFP fluorescence intensity was greater in homozygotes**

**(A-B)** Representative images of (A) NG2DsRed x CX_3_CR1^+/GFP^ and (B) NG2DsRed x CX_3_CR1^GFP/GFP^ tissue showing higher GFP fluorescence intensity in CX_3_CR1^GFP/GFP^ tissue compared to CX_3_CR1^+/GFP^ tissue (images were captured with the same exposure times and brightness and contrast were set at the same level). Dashed boxes in (A) and (B) are magnified in the bottom left corner. **(C)** Quantification of microglia GFP fluorescence intensity in the somatosensory cortex from NG2DsRed x CX_3_CR1^+/GFP^ vs. NG2DsRed x CX_3_CR1^GFP/GFP^ mice. Analysed with an unpaired nonparametric Mann-Whitney test. **(D)** Representative example of individual microglia GFP fluorescence detected from one NG2DsRed x CX_3_CR1^+/GFP^ replicate vs. one NG2DsRed x CX_3_CR1^GFP/GFP^ replicate in the somatosensory cortex. **(E-F)** Quantification of microglia and pericytes in the somatosensory cortex (Bregma -1.5, *n* = 5 per group, two males and three females for NG2DsRed x CX_3_CR1^+/GFP^, three males and two females for NG2DsRed x CX_3_CR1^GFP/GFP^). (E-F) Data compared with unpaired parametric t-test. For panels C, E-F, grey circles represent males and white circles represent females. Data are presented as mean ± SD. ***p* < 0.01.

**
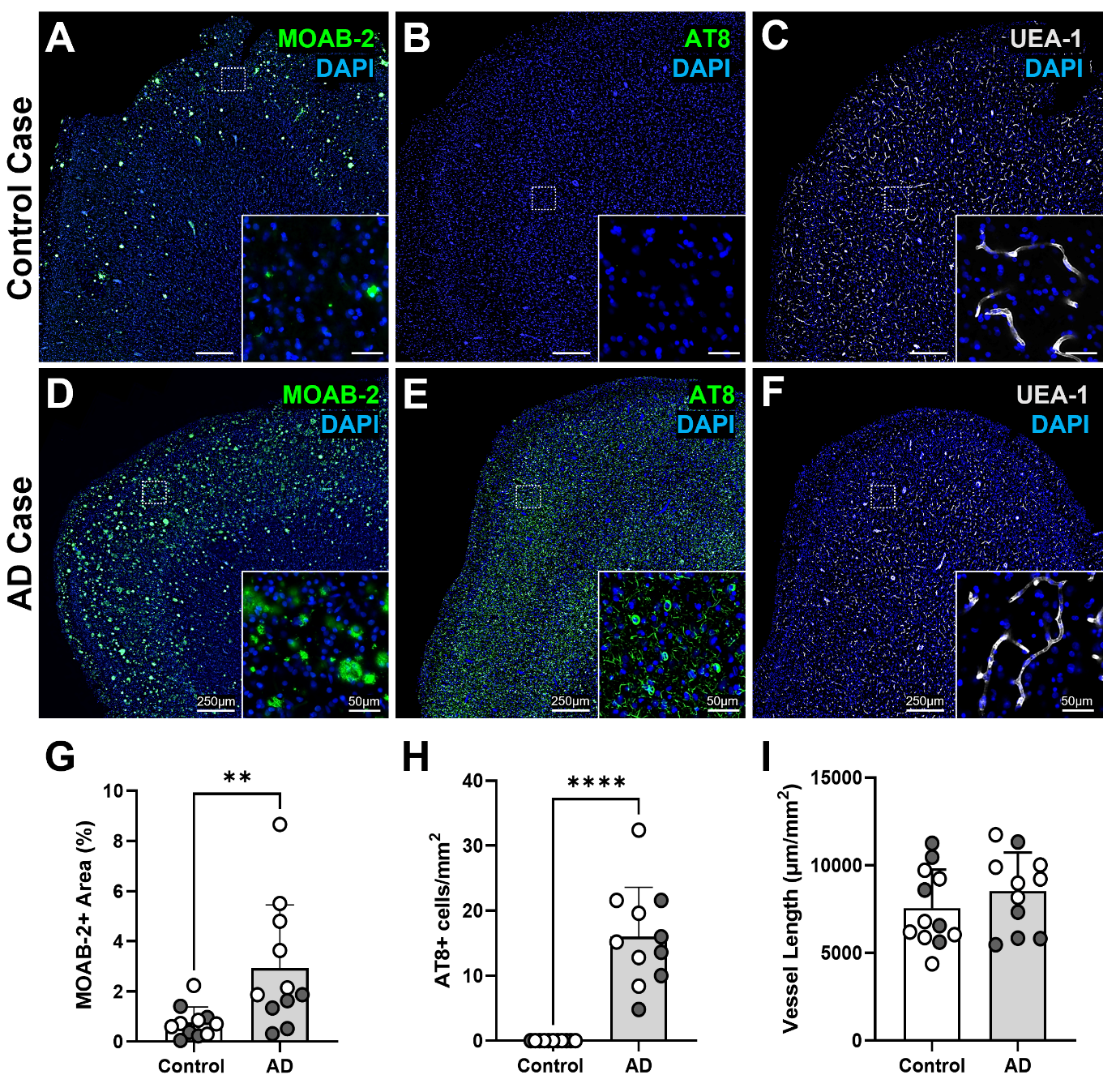
**

**Supplementary Figure 10. Assessment of amyloid pathology, tau pathology and capillaries in the human superior frontal gyrus of AD and control cases**

**(A-F)** Representative images of (A, D) MOAB-2 labelling of Aβ pathology, (B, E) AT8 labelling of NFTs and (C, F) UEA-1 labelling of blood vessels in the SFG of (A-C) control and (D-F) AD cases. Dashed boxes in (A-F) are magnified in the bottom right corner. A is from a 95 y.o. female, B-C is from a 93 y.o. female, D is from an 81 y.o. female, E-F is from a 76 y.o. female. **(G-I)** Quantification of (G) MOAB-2 labelling to determine the percentage area of MOAB-2, (H) AT8 labelling determine the number of AT8 positive cells per mm² and (I) UEA-1 labelling determine capillary length in tissue sections from the SFG in control and AD post-mortem brains (*n* = 11 per group). (G-H) were analysed with an unpaired nonparametric Mann-Whitney test. (I) was analysed with an unpaired parametric t-test. For all graphs, grey circles represent males and white circles represent females. Data are presented as mean ± SD.

**Supplementary Movie 1:** 3D reconstruction of Fig. 1A, derived from a 19 μm z-stack imaged with 1 μm increments.

**Supplementary Movie 2:** 3D reconstruction of Fig. 1B, derived from a 32 μm z-stack imaged with 0.5 μm increments.

**Supplementary Movie 3:** 3D reconstruction of Supplementary Fig. 3, derived from a 21 μm z-stack imaged with 1 μm increments.

**Supplementary Movie 4:** Movie of a 2D 2PLSM image from Fig. 1H, moving through a 101μm z-stack.

**Supplementary Movie 5:** 3D reconstruction of Fig. 1L, derived from an 18 μm z-stack imaged with 0.5 μm increments.

**Supplementary Movie 6:** 3D reconstruction of Fig. 3D, derived from a 23 μm z-stack imaged with 1 μm increments.

**Supplementary Movie 7:** 3D reconstruction of Supplementary Fig. 7A, derived from a 26 μm z-stack imaged with 1 μm increments.

**Supplementary Movie 8:** 3D reconstruction of Supplementary Fig. 7B, a crop of Supplementary Fig. 7A.

**Supplementary Movie 9:** 3D reconstruction of Fig. 4A-B and Supplementary Fig. 7D-E, derived from a 25 μm z-stack imaged with 0.5 μm increments.

**Supplementary Movie 10:** 3D reconstruction of Supplementary Fig. 7E, a crop of Supplementary Fig. 7D.

**Supplementary Movie 11:** 3D reconstruction of Fig. 7A, derived from a 20.5 μm z-stack, imaged with 0.5 μm increments.

**Supplementary Table 1 Extended information on individual human control and AD cases**


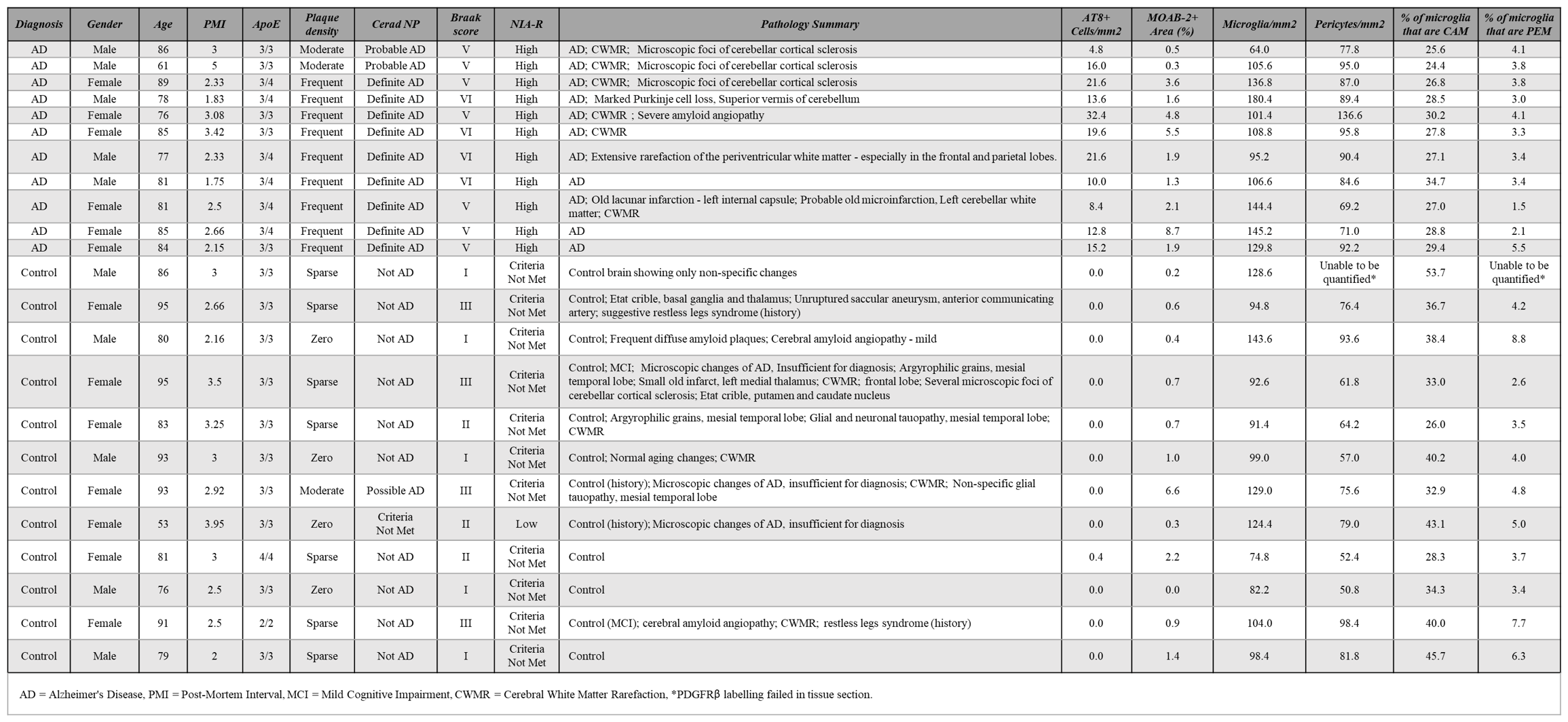


**References**

Allen Reference Atlas. Retrieved from – Mouse Brain [brain atlas]. Available from atlas.brain-map.org.

Arganda-Carreras, I., Fernández-González, R., Muñoz-Barrutia, A., & Ortiz-De-Solorzano, C. (2010). 3D reconstruction of histological sections: Application to mammary gland tissue. *Microsc Res Tech, 73*(11), 1019-1029. doi:10.1002/jemt.20829

Arganda-Carreras, I., Kaynig, V., Rueden, C., Eliceiri, K. W., Schindelin, J., Cardona, A., & Sebastian Seung, H. (2017). Trainable Weka Segmentation: a machine learning tool for microscopy pixel classification. *Bioinformatics, 33*(15), 2424-2426. doi:10.1093/bioinformatics/btx180

Attwell, D., Mishra, A., Hall, C. N., O'Farrell, F. M., & Dalkara, T. (2016). What is a pericyte? *J Cereb Blood Flow Metab, 36*(2), 451-455. doi:10.1177/0271678x15610340

Bonney, S. K., Sullivan, L. T., Cherry, T. J., Daneman, R., & Shih, A. Y. (2022). Distinct features of brain perivascular fibroblasts and mural cells revealed by in vivo two-photon imaging. *J Cereb Blood Flow Metab, 42*(6), 966-978. doi:10.1177/0271678x211068528

Courtney, J.-M., Morris, G. P., Cleary, E. M., Howells, D. W., & Sutherland, B. A. (2021). An Automated Approach to Improve the Quantification of Pericytes and Microglia in Whole Mouse Brain Sections. *eneuro, 8*(6), ENEURO.0177-0121.2021. doi:10.1523/eneuro.0177-21.2021

Courtney, J.-M., Morris, G. P., Cleary, E. M., Howells, D. W., & Sutherland, B. A. (2022). Automated Quantification of Multiple Cell Types in Fluorescently Labeled Whole Mouse Brain Sections Using QuPath. *Bio-protocol, 12*(13), e4459. doi:10.21769/BioProtoc.4459

Morris, G. P., Gowing, E. K., Courtney, J.-M., Coombe, H. E., King, N. E., Rewell, S. S. J., . . . Sutherland, B. A. (2023). Vascular perfusion differs in two distinct PDGFRβ-positive zones within the ischemic core of male mice 2 weeks following photothrombotic stroke. *Journal of Neuroscience Research, 101*(2), 278-292. doi:<https://doi.org/10.1002/jnr.25146>

Watson, C., Paxinos, G., Kayalioglu, G., & Heise, C. (2009). Chapter 16 - Atlas of the Mouse Spinal Cord. In C. Watson, G. Paxinos, & G. Kayalioglu (Eds.), *The Spinal Cord* (pp. 308-379). San Diego: Academic Press.
